# Rumen Microbiota, Predicted Metabolic Pathways, and Fermentation Parameters Exhibit Production-System-Specific Associations in Cattle and Goats

**DOI:** 10.3390/microorganisms14091935

**Published:** 2026-09-01

**Authors:** Chao Cui, Yujie Zheng, Zhiguo Guo, Zhichang Wang, Xiaoyan Zhu, Yalei Cui, Defeng Li, Wen Chen, Yinghua Shi, Boshuai Liu

**Affiliations:** 1 Department of Animal Nutrition and Feed Science, College of Animal Science and Technology, Henan Agricultural University, Zhengzhou 450002, Chinacchenwen@aliyun.com (W.C.); 2 Henan Key Laboratory of Innovation and Utilization of Grassland Resources, Zhengzhou 450002, China; 3 Henan Herbage Engineering Technology Research Center, Zhengzhou 450002, China

**Keywords:** rumen microbiome, flora-typing, short-chain fatty acids, methane metabolism, host–microbiome interaction, cross-species comparison

## Abstract

The specific differences, unique characteristics, and functional linkages of rumen microbiota and their metabolic pathways across different ruminant species remain poorly understood. To investigate these cross-species relationships, 16 Holstein cows, 28 Simmental crossbred cattle, and 15 Boer goats were fed under standardized conditions for 97 days. Rumen microbial composition and function were evaluated using 16S rRNA gene sequencing and PICRUSt. Results revealed distinct, host-specific microbial architectures. Holstein cows exhibited an overall enrichment of the phylum *Proteobacteria* and ABC transporter pathways. Notably, their micro-ecosystem was dominated by the *Succinivibrionaceae_UCG-001* flora type, which is computationally associated with propionate synthesis for milk production and is predicted to potentially contribute to altered methane metabolism. In contrast, Simmental cattle were characterized by *Succinivibrionaceae_UCG-002* and computationally upregulated glycolysis/gluconeogenesis pathways, which are associated with volatile fatty acid conversion for energy deposition. Furthermore, Boer goats harbored a unique fiber-adapted ecosystem exclusively enriched with the *norank_f_Bacteroidales_BS11_gut_group* and *Lachnospiraceae_ND3007_group*, correlating with butyrate production and maintaining a classic acetate-type fermentation profile, while exhibiting an elevated predicted genomic potential for methane production based on functional profiling. This study demonstrates that rumen microbiota, metabolic pathways, and fermentation parameters form a highly coordinated network shaped by host phylogeny, energy allocation, and specific diets, providing a theoretical basis for targeted microbiome modifications to improve animal health and feed utilization.

## 1. Introduction

In the face of dual global challenges—meeting the surging demand for high-quality animal protein and mitigating greenhouse gas emissions from the livestock sector—enhancing feed conversion efficiency and optimizing metabolic homeostasis in ruminants have emerged as central imperatives in modern agricultural science. The remarkable ability of ruminants to efficiently utilize coarse forage, which is indigestible to humans, relies entirely on their highly evolved and complex rumen microecological system [1]. The vast microbial community residing within the rumen undergoes highly intricate, synergistic fermentation to degrade plant polysaccharides, such as cellulose and hemicellulose, into volatile fatty acids (VFAs) and microbial crude protein (MCP). These end-products supply 70% to 80% of the daily energy required for host maintenance and production, serving as the fundamental drivers determining the yield and quality of animal products (milk and meat) [2,3]. Consequently, precisely deciphering and selectively targeting the rumen microbiome has become a critical breakthrough point for achieving precision nutrition and sustainable development in ruminant production [3].

Over the past decades, multi-omics research has extensively corroborated that exogenous dietary composition is the primary driver shaping the succession of the rumen microbial community [4]. It is hypothesized that under modern intensive farming systems, the rumen microecology is not solely dictated by diet; rather, it may be the consequence of long-term interactions between exogenous nutritional inputs and the host’s endogenous traits [5,6]. Through physiological mechanisms including unique chewing and rumination patterns, salivary buffering capacity, and digesta passage rates, the host—in tandem with its corresponding species-specific dietary regimen—exerts a continuous bidirectional selection pressure, driving the co-evolution of the rumen microbiome [7].

Holstein cows, Simmental crossbred cattle, and Boer goats represent three of the most economically significant ruminant species worldwide. These three archetypal livestock species were selected because they epitomize extreme physiological divergence in energy allocation patterns and are managed under distinctly different standardized feeding systems in practical agricultural production. It must be explicitly noted early on that these animals represent distinct, integrated farming systems rather than controlled, isolated genetic contrasts. Lactating Holstein cows remain in a hypermetabolic state, requiring nutrient-dense diets and relying heavily on the rumen to generate copious amounts of propionate—the primary precursor for gluconeogenesis—to synthesize lactose and sustain high milk yields [8]. Conversely, Simmental beef cattle are tailored for rapid skeletal muscle accretion and fat deposition, demanding exceptionally high efficiencies in dietary energy conversion and nitrogen retention [9]. Meanwhile, Boer goats, as typical small ruminants, exhibit robust tolerance to coarse forage, and their distinct ruminal milieu (e.g., higher average pH levels) fosters fermentation and metabolic profiles that diverge sharply from those of large bovines. Consequently, it must be acknowledged that physiological stages, production objectives, age, and dietary inputs are analyzed here as combined, species-specific production systems rather than isolated variables.

Previous studies suggest that the genus Prevotella, the family *Succinivibrionaceae*, and certain specific *bacteroidetes* (such as the BS11 gut group) play pivotal roles in host substrate degradation and methane mitigation [10]. However, current microecological investigations into the ruminant rumen microbiome predominantly focus on longitudinal evaluations of different dietary formulations within a single species. Scant research has approached this from a macroscopic perspective of “systems agroecology” to systematically and transversely evaluate the microbial differentiation among these three archetypal livestock species under their respective standardized production states. Under their typical large-scale farming and dietary paradigms, the precise systemic discrepancies in ruminal “flora-typing”, profound metabolic pathway potentials, and fermentation phenotypes remain an unresolved enigma, lacking comprehensive and robust cross-species comparative data.

Addressing this critical knowledge gap, the present study hypothesized that due to fundamental differences in long-term selection directions (dairy vs. beef), phylogenetic characteristics (large Bovinae vs. small Caprinae), and their consequent species-specific nutritional supply models, the rumens of Holstein cows, Simmental crossbred cattle, and Boer goats under typical production conditions harbor highly system-specific core flora types. Furthermore, this microecological heterogeneity—synergistically driven by host physiology and specific diets—will profoundly manifest in their foundational metabolic networks and ultimate fermentation phenotypes.

To systematically test this hypothesis, the current study utilized Holstein cows, Simmental crossbred cattle, and Boer goats at typical production stages as research models. Under their respective standardized husbandry and dietary feeding regimens, a cross-species transverse comparison was conducted integrating 16S rRNA gene amplicon deep sequencing with physicochemical analyses. First, the study accurately assessed and characterized the structural differences and specific core flora-typing profiles of the rumen microbial communities across the three hosts. Building upon this, the PICRUSt predictive algorithm coupled with the KEGG database was employed to deeply excavate the functional potential of these divergent micro-ecosystems regarding substrate transport and energy retention pathways. Ultimately, by constructing a multidimensional correlation network encompassing the core microbiota, functional metabolic pathways, and fermentation phenotypic parameters, we comprehensively delineated the interactive relationships between the microecological communities and their respective production systems. The findings of this study will not only unveil the baseline characteristics and differentiation patterns of ruminal milieus among diverse ruminants in real-world large-scale production but also provide pivotal theoretical frameworks and empirical data to support the future development of targeted microbiome manipulation technologies and the formulation of species-specific nutritional strategies aimed at simultaneously enhancing productivity and mitigating methane emissions.

## 2. Materials and Methods

### 2.1. Animals, Diets, and Experimental Design

This study was conducted at three commercial breeding facilities in Zhumadian City, Henan Province, China. A total of 59 animals were selected, comprising 16 mid-lactation Holstein cows (parity = 2–3, average milk yield = 26 kg/d), 28 male Simmental crossbred cattle (age = 15–17 months, body weight = 497–507 kg), and 15 male Boer goats (age = 5 months, body weight = 37–41 kg). The unequal sample sizes reflect the maximum number of compliant animals available at each commercial facility during the trial period; however, post-hoc power analyses confirmed these group sizes retained sufficient statistical power to detect robust structural differences in the microbiome. All animals had free access to clean drinking water throughout the experimental period. Within each farm, conspecific animals were fed species-specific standardized diets formulated to meet their unique nutritional requirements, which minimized environmental and nutritional confounding effects. Full diet compositions and nutritional profiles are provided in Supplementary Tables S1–S3, where the precise forage-to-concentrate ratios and estimated dry matter intake values for each group are documented in detail. All enrolled animals were clinically healthy and had no exposure to antibiotics for at least three months prior to sample collection. Due to the commercial nature of the breeding facilities, each livestock category was housed at a separate farm and supplied with its exclusive diet. Under this experimental design, host phylogeny, farm environment, feeding management and diet composition were fully confounded variables. As such, the observed rumen microbial differences reflect distinctions across the whole production system and cannot be attributed solely to host evolutionary lineage.

### 2.2. Sample Collection and Parameter Measurement

The feeding trial lasted for 97 days (7-day adaptation and 90-day main trial). Animals were fed twice daily at 08:00 and 17:00. Rumen fluid samples were collected on the final day, 2 h after the morning feeding. Sampling was performed using a customized esophageal rumen tube. To prevent saliva contamination, the initial 1.0 L of extracted rumen fluid was discarded, and the subsequent 0.5 L was collected. Immediately after collection, a sub-sample (approximately 5 mL) was vigorously homogenized and snap-frozen in liquid nitrogen (−80 °C) for subsequent 16S rRNA gene sequencing.

The remaining rumen fluid was filtered through four layers of sterile cheesecloth. Ruminal pH was recorded immediately using a portable pH meter (testo 206, testo AE & Co. KGaA, Lenzkirch, Germany). For the determination of volatile fatty acids (VFAs) and ammonia nitrogen (NH_3_-N), fluid aliquots were mixed with 25% (*w*/*v*) metaphosphoric acid and centrifuged at 3000× *g* for 15 min at 4 °C. NH3-N concentration was determined using a standard colorimetric method. VFA profiles were quantified via gas chromatography (GC-2014, Shimadzu Corporation, kyoto, Japan) equipped with a flame ionization detector (FID) and a capillary column (e.g., DB-FFAP, 30 m × 0.32 mm × 0.25 μm). Quantification was performed using 2-ethylbutyric acid as the internal standard, with an exact sample injection volume of 1 µL. Nitrogen was used as the carrier gas at a flow rate of 2.0 mL/min. The temperatures of the injector and detector were set at 250 °C and 250 °C, respectively. The oven temperature program was set as follows: initial temperature of 120 °C held for 3 min, increased to 180 °C at a rate of 10 °C/min, and held at 180 °C for 3 min. These procedures followed the standardized analytical protocols described in recent rumen studies [4].

This study employed custom-made esophageal-rumen tubes. While the initial 1.0 L of extracted rumen fluid was discarded to minimize saliva contamination, it is acknowledged that this sampling methodology can still inherently influence pH measurements and the relative abundance of specific microbial taxa compared to rumen cannula sampling; however, such saliva contamination exerts a negligible differential impact on the molar proportions of volatile fatty acids.

### 2.3. DNA Extraction and 16S rRNA Amplicon Sequencing

Total microbial genomic DNA was extracted from the rumen fluid samples using the E.Z.N.A.^®^ DNA Kit (Omega Bio-tek, Norcross, GA, USA). To rigorously monitor and account for potential environmental or reagent-based contamination, blank controls utilizing sterile nuclease-free water in place of rumen fluid were processed in parallel throughout the entire DNA extraction procedure. The blank samples and negative PCR controls were sequenced alongside biological samples in the subsequent bioinformatic pipeline to identify and remove potential background contaminants. Subsequently, DNA concentration and integrity were evaluated using a NanoDrop 2000 spectrophotometer (Thermo Fisher Scientific, Wikming, DE, USA) and 2% agarose gel electrophoresis, respectively, following established rumen microbiome protocols [11].

The hypervariable V3-V4 region of the bacterial 16S rRNA gene was amplified using the universal primer pair 338F (5′-ACTCCTACGGGAGGCAGCAG-3′) and 806R (5′-GGACTACHVGGGTWTCTAAT-3′). The resulting PCR products were purified using an Axygen DNA Gel Extraction Kit (Corning, NY, USA) and quantified. Equimolar amounts of purified amplicons were pooled to construct paired-end libraries. Sequencing was performed on an Illumina MiSeq platform (Majorbio, Shanghai, China).

### 2.4. Bioinformatic Processing

Bioinformatic analyses of the raw sequencing data were conducted as outlined by Li et al. [12]. Paired-end reads were merged using FLASH (v.1.2.11) and subjected to rigorous quality filtering using fastp (version 0.19.6). Specifically, sequences were truncated at any site receiving an average quality score of <20 over a 50 bp sliding window. Reads containing ambiguous bases (N), harboring more than zero nucleotide mismatches in the barcode or up to two mismatches in the primers, or shorter than 50 bp after trimming were completely discarded to ensure high-quality data for downstream analysis. Following the consensus best practices for 16S microbiome studies [13], high-quality sequences were denoised to generate Amplicon Sequence Variants (ASVs) using DADA2 v.1.16, and chimeric sequences were removed. The use of ASVs provides single-nucleotide resolution and ensures consistency with modern high-resolution microbiome studies, thereby maintaining horizontal comparability between groups.

Representative sequences for each ASV were taxonomically annotated against the continuously updated SILVA database [14] using the RDP Classifier (v2.13). Alpha diversity indices (Shannon, Simpson, ACE, Chao1) were calculated using Mothur (v.1.30.2). Beta diversity was visualized via Principal Coordinate Analysis (PCoA). Analysis of molecular variance (AMOVA) and linear discriminant analysis effect size (LEfSe) were utilized to assess structural differences and identify discriminative taxa among groups [15]. Functional profiles of the rumen microbiota were predicted from ASV abundance data using PICRUSt2, and differential metabolic pathways were annotated according to the KEGG database at Levels 1 and 3, consistent with recent ruminant metabolic studies [15]. It is important to note that the reference genome for rumen microbiota is currently incomplete, and PICRUSt can only predict the potential metabolic capabilities of the microbial community but cannot represent the actual transcriptome and metabolic activity within the body. When interpreting the results of metabolic pathways, one must take into account the limitations of this method. All visualizations in this study were created using R software (version 4.0.2).

### 2.5. Statistical Analysis

Statistical analyses were conducted using IBM SPSS Statistics 23.0 and R software (v.4.0.2). Prior to intergroup comparisons, the Shapiro–Wilk test and Levene’s test were used to verify data normality and homogeneity of variances, respectively. One-way analysis of variance (ANOVA) was adopted for datasets satisfying parametric assumptions, while the non-parametric Kruskal–Wallis H test was applied for non-normal data, followed by Wilcoxon rank-sum tests for pairwise comparisons. All *p*-values from multiple comparisons, including pairwise group differences and all Spearman’s rank correlation matrices (genus–pathway and genus/phylum–fermentation parameter pairs), were adjusted via the false discovery rate (FDR) method to minimize the risk of false-positive correlations. The threshold for statistical significance was set at *p* < 0.05 throughout the study. Furthermore, the unequal sample sizes across groups (16 Holstein cows, 28 Simmental crossbred cattle, and 15 Boer goats) constitute a study-wide statistical limitation that must be considered when interpreting absolute estimations of diversity indices and differential abundance.

### 2.6. Data Availability

The raw sequencing data have been deposited in the NCBI Sequence Read Archive (SRA) database under the accession numbers SRP298528, SRP291981, SRP298526, and SRP299548.

## 3. Results

### 3.1. Sequencing Output and α-Diversity of the Rumen Microbiota

**Figure 1:**
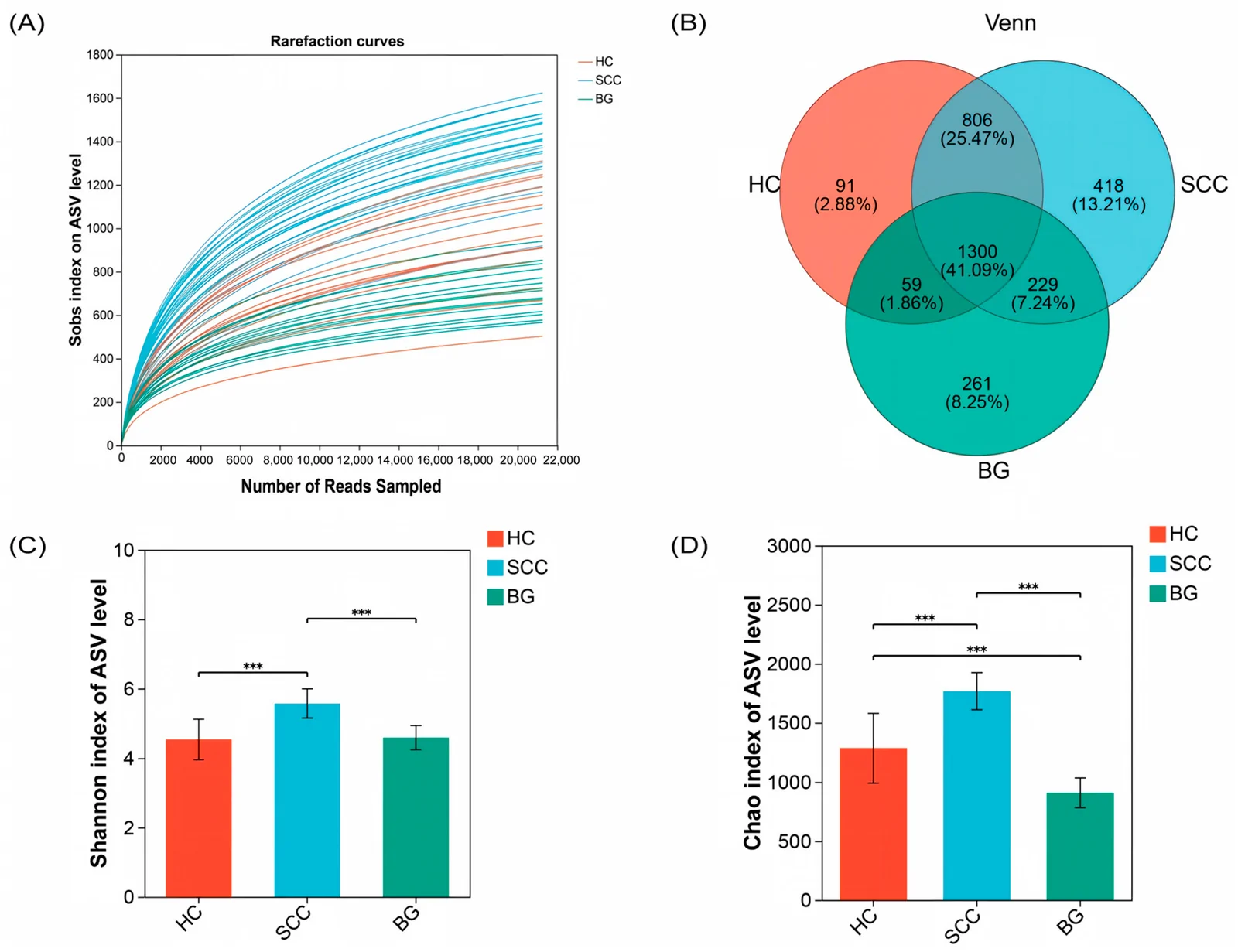
Rarefaction curves (**A**) and Venn diagrams (**B**) of rumen microbiota of Holstein cows (HC), Simmental crossbred cattle (SCC), and Boer goats (BG) at the ASV level. (**C**,**D**) Analysis of differences between HC, SC, and BG rumen microbial Shannon index and Chao index groups. Highly significant differences between pairs of selected samples are indicated, *** *p* < 0.001. The horizontal coordinate is the group name, and the vertical coordinate is the average of the indices for each group.

The rumen microbiota of Holstein cows, Simmental crossbred cattle, and Boer goats were profiled via deep sequencing of the 16S rRNA gene V3-V4 region. A total of 2,752,177 high-quality sequence reads were obtained across all samples (Figure 1A). Denoising of the high-quality sequence reads yielded 2256, 2753, and 1849 Amplicon Sequence Variants (ASVs) in the rumens of Holstein cows, Simmental crossbred cattle, and Boer goats, respectively. Venn diagram analysis revealed that 806 ASVs were shared between Holstein cows and Simmental cattle, 59 between Holstein cows and Boer goats, and 229 between Simmental cattle and Boer goats (Figure 1B).

Alpha-diversity analysis demonstrated significant variations among the three ruminant groups. Specifically, the Shannon and Chao1 indices of Simmental crossbred cattle were significantly higher than those of both Holstein cows and Boer goats. Furthermore, these indices in Holstein cows were also significantly higher compared to Boer goats (Figure 1C,D). These findings indicate that the ruminal microbial community in Simmental cattle possesses the highest richness and diversity, while Boer goats exhibit the lowest, with Holstein cows and Simmental cattle sharing more similarities in their microbiota. Although the rarefaction curves of all groups approached plateaus, the unequal sample sizes across groups (16 Holstein cows, 28 Simmental crossbred cattle, and 15 Boer goats) constitute a statistical limitation, which may introduce minor biases into the absolute estimation of alpha diversity indices.

### 3.2. Taxonomic Composition of the Rumen Microbiota

At the phylum level, *Bacteroidetes*, *Firmicutes*, and *Proteobacteria* were identified as the predominant taxa across all three ruminant species. *Bacteroidetes* accounted for the largest proportion of the total ruminal microbiota in Boer goats (60.65%), followed by Simmental cattle (58.18%) and Holstein cows (45.81%). *Firmicutes* constituted 31.21%, 29.79%, and 28.30% of the ruminal microbiota in Holstein cows, Simmental cattle, and Boer goats, respectively. Conversely, *Proteobacteria* was highly enriched in Holstein cows (18.44%), compared to Simmental cattle (5.50%) and Boer goats (4.90%) (Supplementary Figure S1A). At the genus level, *Prevotella_1*, *Succiniclasticum*, and *norank_f_Bacteroidales_BS11_gut_group* were the dominant genera. Prevotella_1 represented 30.24%, 35.56%, and 22.76% of the ruminal microbiota in Holstein cows, Simmental cattle, and Boer goats, respectively. *Succiniclasticum* was most abundant in Holstein cows (11.24%), while the *norank_f_Bacteroidales_BS11_gut_group* was highly enriched in Boer goats (11.97%) (Supplementary Figure S1B).

**Figure 2:**
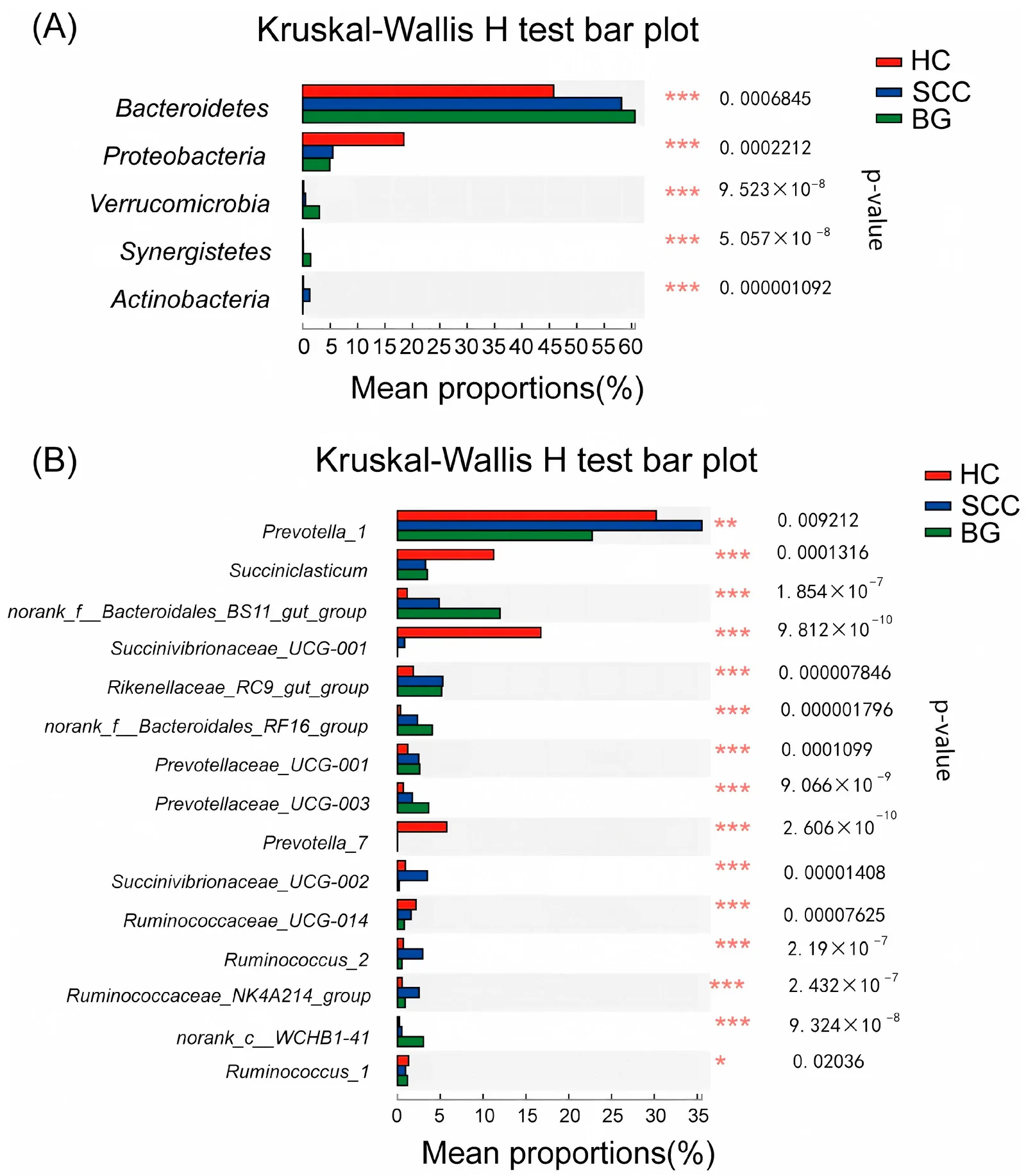
Differences in rumen microbiota among Holstein cows (HC), Simmental crossbred cattle (SCC), and Boer goats (BG) at phylum (**A**) and genus (**B**) levels. The *y*-axis represents the species name at a certain taxonomic level, the *x*-axis represents the average relative abundance in different groups of species, and the columns of different colors represent the different ruminant groups. Values on the right of the plot are *p*-values indicating significance, * *p* < 0.05, ** *p* < 0.01, *** *p* < 0.001.

Further comparative analysis (Figure 2A,B) revealed distinct taxonomic variations among the groups. At the phylum level, the relative abundance of *Proteobacteria* was significantly higher in Holstein cows than in Simmental cattle and Boer goats (*p* < 0.001), whereas *Verrucomicrobia* and *Synergistetes* were considerably diminished in Holstein cows compared to Boer goats. The abundance of *Bacteroidetes* was significantly lower in Holstein cows than in the other two groups, while *Actinobacteria* was more abundant in both bovine species compared to Boer goats.

At the genus level, Holstein cows exhibited significantly higher relative abundances of *Succiniclasticum*, *Succinivibrionaceae_UCG-001*, and *Prevotella_7* compared to both Simmental cattle and Boer goats (*p* < 0.001). Additionally, *Ruminococcaceae_UCG-014* was significantly elevated in Holstein cows versus Boer goats (*p* < 0.001). In contrast, Simmental cattle showed significantly higher abundances of *Succinivibrionaceae_UCG-002*, *Ruminococcus_2*, and *Ruminococcaceae_NK4A214_group* compared to both Holstein cows and Boer goats (all *p* < 0.01 or *p* < 0.001). Furthermore, Boer goats were characterized by significantly higher relative abundances of *norank_f_Bacteroidales_BS11_gut_group*, *Prevotellaceae_UCG-003*, *norank_c_WCHB1-41DC*, and *Lachnospiraceae_ND3007_group* compared to the two bovine groups (all *p* < 0.01 or *p* < 0.001).

To ensure statistical rigor across numerous pairwise comparisons among microbial taxa and KEGG pathways, *p*-values were adjusted using the False Discovery Rate (FDR) method, and significance was declared at an FDR-adjusted *p* < 0.05 (q-value < 0.05).

### 3.3. Beta Diversity and Structural Differences of the Rumen Microbiota

**Figure 3:**
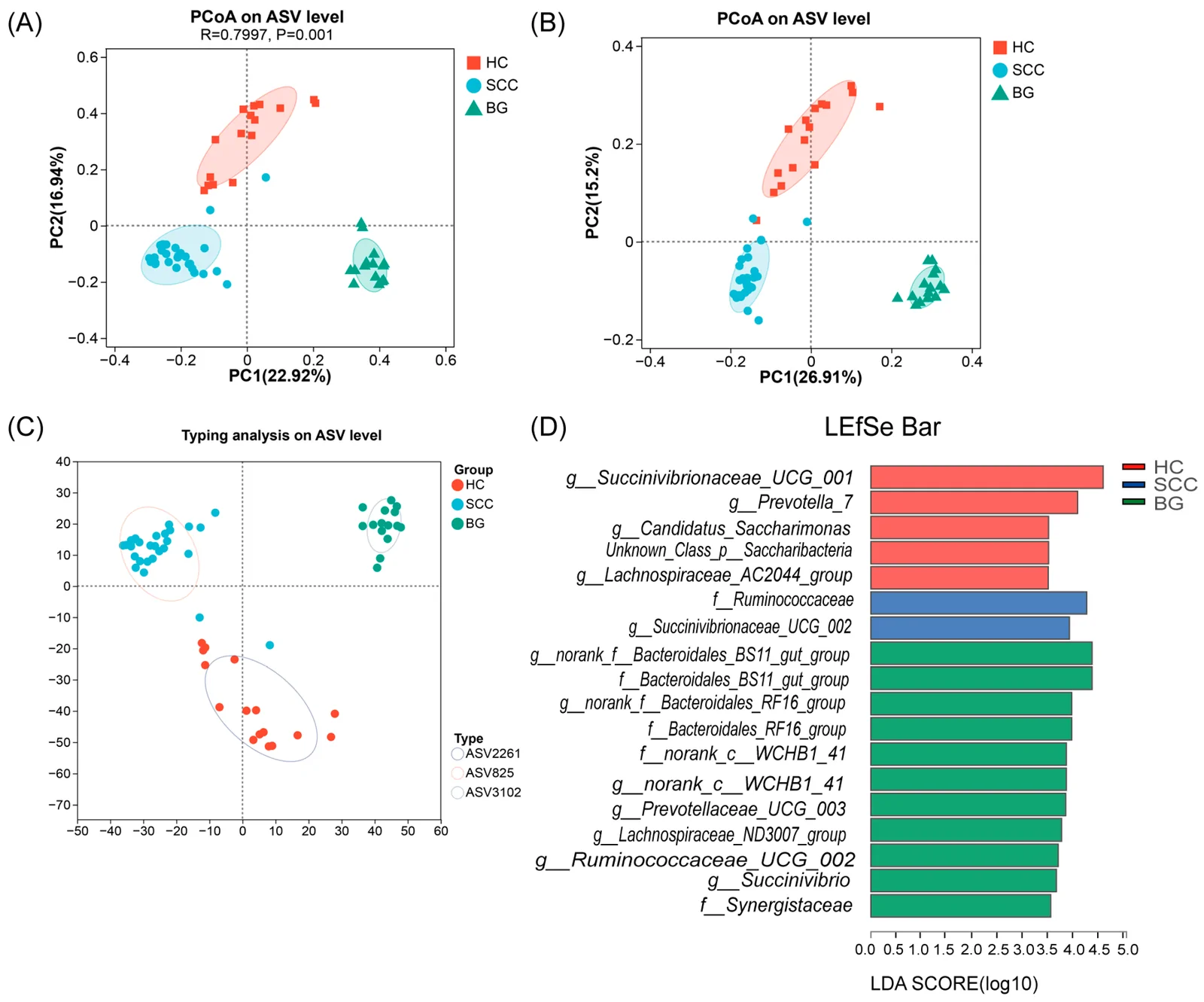
PCoA and typing analysis of rumen microbiota of Holstein cows (HC), Simmental crossbred cattle (SCC), and Boer goats (BG) at the ASV level. Rumen microbiota were analyzed by PCoA based on Bray–Curtis distance (**A**), by PCoA based on unweighted_unifrac distance (**B**), and by typing analysis (**C**). (**D**) LEfSe multistage species difference discriminant analysis map of rumen microbiota of HC, SC, and BG (LDA threshold is 3.5).

Beta diversity was evaluated via Principal Coordinate Analysis (PCoA) based on Bray–Curtis and unweighted UniFrac distance matrices (Figure 3A,B). The PCoA plots demonstrated that the ruminal microbial samples from Holstein cows and Simmental crossbred cattle clustered relatively close to each other, indicating similarities in their community composition and structure. In sharp contrast, the rumen microbiota of Boer goats separated distinctly from those of both Holstein cows and Simmental crossbred cattle along the principal coordinate axes, revealing substantial structural divergence. We performed PCoA visualization based on Bray–Curtis and unweighted UniFrac distance matrices, and a PERMANOVA test was applied to quantify the differences in microbial community structure among groups. The results demonstrated statistically significant intergroup divergence in community structure (R = 0.7997, *p* = 0.001).

### 3.4. Community Typing (Flora-Typing) of the Rumen Microbiota

A flora-typing analysis (Figure 3C) was conducted to cluster the ruminal microbiota of the three species into distinct enterotypes. The results revealed that the microbial community in Holstein cows was predominantly driven by *Succinivibrionaceae_UCG-001* (87.50%) and *Succinivibrionaceae_UCG-002* (12.50%). Similarly, the microbiota of Simmental crossbred cattle clustered into *Succinivibrionaceae_UCG-002* (96.43%) and *Succinivibrionaceae_UCG-001* (3.57%). Conversely, the ruminal microbiota of Boer goats was exclusively classified into the *norank_f_Bacteroidales_BS11_gut_group* type (100.00%). The partitioning around medoids (PAM) algorithm was adopted for flora-typing clustering in the present study, and the Calinski–Harabasz index was used to objectively select the optimal number of clusters, providing solid methodological basis for the classification criteria of flora typing.

### 3.5. Identification of Differential Microbial Taxa via LEfSe Analysis

Linear discriminant analysis (LDA) effect size (LEfSe) was employed to identify robust microbial biomarkers that differed significantly among the groups (LDA score threshold > 3.5) (Figure 3D). The analysis revealed that the rumen of Simmental crossbred cattle was significantly enriched with *Succinivibrionaceae_UCG-002* at the genus level. In Holstein cows, *Succinivibrionaceae_UCG-001* and *Prevotella_7* emerged as the major differential biomarkers. Meanwhile, the rumen of Boer goats was significantly enriched with *norank_f_Bacteroidales_BS11_gut_group*, *norank_f_Bacteroidales_RF16_group*, and *Lachnospiraceae_ND3007_group*.

### 3.6. Predictive Functional Profiling of the Rumen Microbiome (KEGG Pathways)

**Figure 4:**
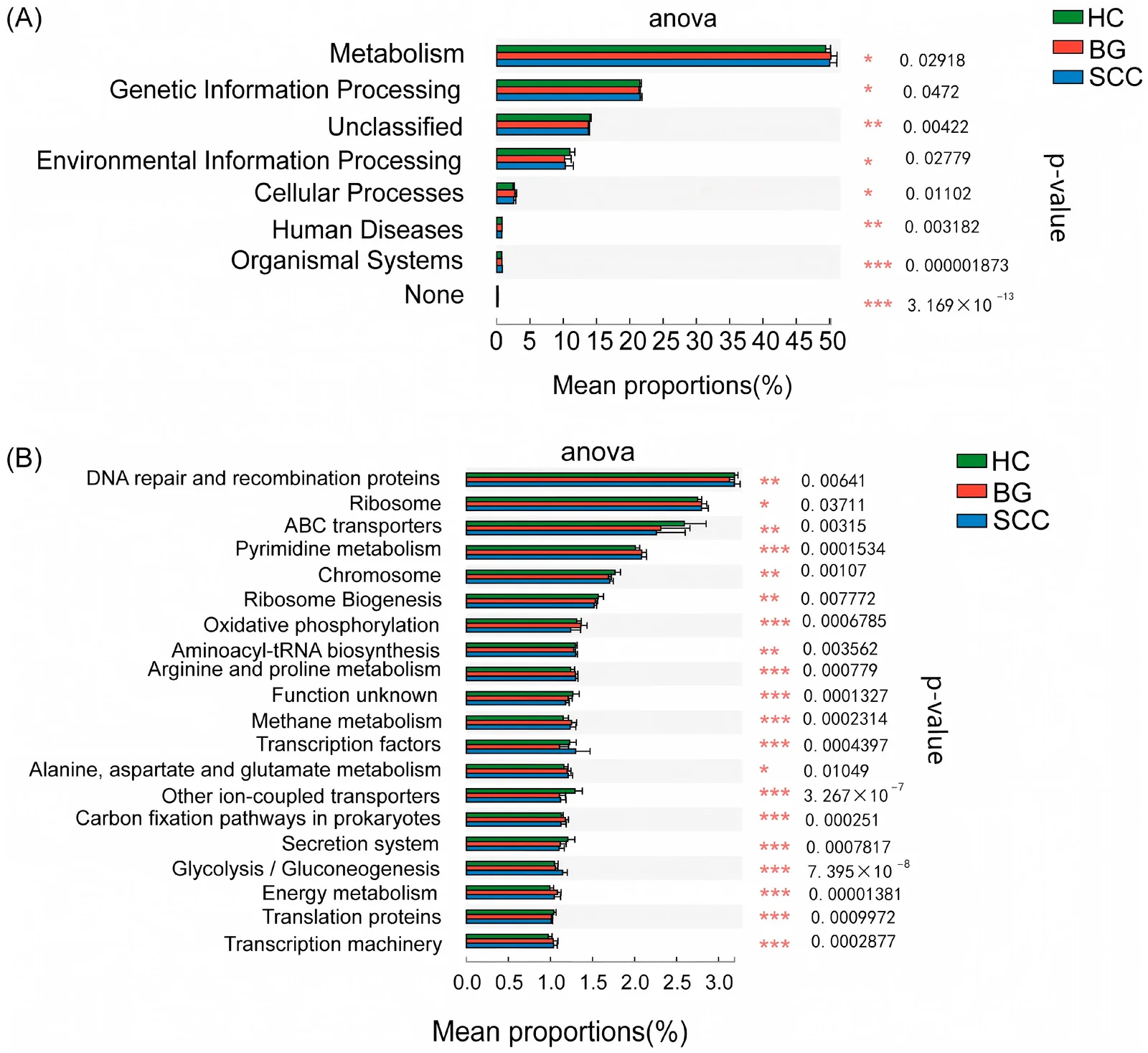
Functional prediction of rumen microbiota at genus level in Holstein cows (HC), Simmental crossbred cattle (SCC), and Boer goats (BG) using KEGG Pathway Level 1 (**A**) and Level 3 (**B**). The *y*-axis indicates the name of a metabolic pathway, the *x*-axis indicates the average relative abundance in different groupings of species, and the different colored columns indicate the different ruminant groupings. Values on the right are *p*-values indicating significance, * *p* < 0.05, ** *p* < 0.01, *** *p* < 0.001.

The functional potential of the rumen microbiota was predicted using PICRUSt based on the KEGG database. At KEGG Level 1, the relative abundance of pathways related to cellular processes was significantly lower in both Holstein cows and Simmental cattle compared to Boer goats (*p* < 0.01) (Figure 4A and Figure S2A–C).

At KEGG Level 3 (Figure 4B and Figures S3–S5), the prominent metabolic pathways across all three species included ATP-binding cassette (ABC) transporters, oxidative phosphorylation, methane metabolism, carbon fixation pathways in prokaryotes, glycolysis/gluconeogenesis, and energy metabolism. Specifically, the abundance of ABC transporters was significantly higher in Holstein cows than in Simmental cattle (*p* < 0.001) and Boer goats (*p* < 0.05). Conversely, the glycolysis/gluconeogenesis pathway was significantly enriched in Simmental cattle compared to Holstein cows and Boer goats (*p* < 0.001). The abundances of oxidative phosphorylation and several prokaryotic carbon fixation pathways were significantly lower in both bovine species compared to Boer goats. Furthermore, pathways associated with methane metabolism and overall energy metabolism were considerably downregulated in the rumen of Holstein cows relative to Simmental cattle (*p* < 0.01) and Boer goats (*p* < 0.001).

### 3.7. Rumen Fermentation Parameters

The ruminal fermentation characteristics of the three ruminant species are summarized in Table 1. The ruminal pH of Holstein cows was significantly higher than that of Simmental cattle (*p* < 0.05); however, both bovine species exhibited significantly lower ruminal pH values compared to Boer goats (*p* < 0.05). Additionally, the ruminal NH3-N concentration in Holstein cows was significantly lower than those observed in Simmental cattle and Boer goats (*p* < 0.05).

Regarding the VFA profiles, the molar proportion of acetate in Holstein cows was significantly higher than in Boer goats (*p* < 0.05), while Boer goats had a significantly higher acetate proportion compared to Simmental cattle (*p* < 0.05). The proportion of propionate was highest in Simmental cattle (*p* < 0.05 vs. Holstein cows), and Holstein cows exhibited significantly higher propionate levels than Boer goats (*p* < 0.05). Consequently, the acetate-to-propionate ratio was significantly higher in Boer goats compared to the bovine groups (*p* < 0.05), and this ratio was also significantly higher in Holstein cows than in Simmental cattle (*p* < 0.05).

### 3.8. Correlation Between Specific Rumen Genera and KEGG Level 3 Metabolic Pathways

Spearman correlation analyses were performed to explore the relationships between key ruminal genera and KEGG Level 3 functional pathways (Supplementary Figures S6–S8). In Holstein cows, *Succinivibrionaceae_UCG-001* and *Prevotella_7* exhibited significant positive correlations with methane metabolism, energy metabolism, and prokaryotic carbon fixation pathways, but were negatively correlated with ABC transporters. In Simmental crossbred cattle, *Succinivibrionaceae_UCG-002* was positively correlated with ABC transporters, methane metabolism, oxidative phosphorylation, energy metabolism, and nitrogen metabolism. Conversely, *Prevotella_1* showed negative correlations with these exact pathways. In Boer goats, the *norank_f_Bacteroidales_BS11_gut_group* was positively correlated with ABC transporters, and the *Lachnospiraceae_ND3007_group* showed positive associations with ABC transporters, methane metabolism, oxidative phosphorylation, prokaryotic carbon fixation, and energy metabolism.

### 3.9. Interactions Among Ruminal Microbiota, Metabolic Pathways, and Fermentation Parameters

Further Spearman correlation analyses were utilized to link ruminal microbiota (at the phylum and genus levels) and KEGG pathways with actual fermentation parameters (Supplementary Figure S9). *Firmicutes* was positively correlated with nitrogen metabolism, carbon fixation, methane metabolism, fatty acid metabolism, and fatty acid biosynthesis. *Bacteroidetes* showed positive associations with NH3-N and butyrate but was negatively correlated with fatty acid metabolism and ABC transporters.

At the genus level, *Prevotella_1* exhibited positive correlations with nitrogen metabolism, carbon fixation, energy metabolism, acetate, and propionate but was negatively correlated with butyrate, pH, and the acetate-to-propionate ratio. *Prevotella_7* and *Succinivibrionaceae_UCG-001* were positively associated with the acetate-to-propionate ratio, whereas *Prevotella_7* correlated negatively with absolute acetate, propionate, and energy metabolism. *Succinivibrionaceae_UCG-002* was positively correlated with acetate and propionate but negatively with pH and the acetate-to-propionate ratio. Lastly, *both norank_f_Bacteroidales_BS11_gut_group* and *Lachnospiraceae_ND3007_group* showed positive correlations with butyrate and negative correlations with absolute acetate and propionate levels.

## 4. Discussion

The symbiotic consortium residing within the ruminant gastrointestinal tract represents a canonical model of mammalian evolutionary ecology, where the host’s internal milieu and exogenous nutritional inputs intricately cooperate to govern microbial succession [16]. In modern intensive production systems, this evolutionary dynamic is profoundly accelerated by continuous anthropogenic selection pressures, which have established extreme physiological divergence in energy allocation patterns between dairy, beef, and small ruminant phenotypes [17]. The distinct spatial segregation and minimal topological overlap observed among the ruminal microbial structures of the three livestock cohorts in beta-diversity space provide compelling empirical evidence that host phylogeny, selection history, and species-specific dietary paradigms exert a continuous, bidirectional selective filter that drives micro-ecological niche differentiation [18]. The absolute partitioning of the Boer goat micro-ecosystem from the two bovine cohorts underscores a deep-seated biological barrier governing small Caprinae versus large Bovinae, rooted in divergence of anatomical volumes, mastication-induced rumination kinetics, and salivary buffering capacities [19]. More crucially, by bypassing longitudinal evaluations of isolated dietary variables, this systems-agroecology approach uncovers a highly system-specific “flora-typing” or enterotype-like paradigm that characterizes each production system at baseline. The monotypic clustering of the Boer goat ruminal community exclusively under the uncultured *norank_f_Bacteroidales_BS11_gut_group* flora type underscores a specialized evolutionary adaptation toward extracting energy from complex structural polysaccharides and refractory hemicelluloses [20]. This specialized cellulolytic niche is biochemically sustained by the elevated baseline pH characteristic of the caprine ruminal environment, which offers a highly buffered, stable habitat insulated from the episodic subacute ruminal acidosis frequently encountered in intensively managed cattle [21]. Conversely, the micro-ecological bifurcation observed between the two bovine species, despite their close phylogenetic proximity and shared exposure to concentrate-dense inputs, represents a fine-tuned functional divergence at the genus level [22]. The numerical dominance of *Succinivibrionaceae_UCG-001* in Holstein cows appears consistent with the high-flux, hypermetabolic state required to sustain competitive milk yields, whereas the enrichment of *Succinivibrionaceae_UCG-002* in Simmental crossbred cattle may be associated with maximizing nitrogen retention and net energy conversion for skeletal muscle hypertrophy. These results collectively demonstrate that ruminal enterotypes are not rigidly locked by host phylogeny alone; rather, they emerge as a plastic, malleable state at the intersection of host physiological energy partitioning and exogenous nutritional supply models [23].

This structural divergence at the macroscale is intrinsically linked to the internal complexity and ecological niche widths within each ruminal habitat, as precisely mirrored by the hierarchical gradient in alpha-diversity indices, which peaked in beef cattle, followed by dairy cows, and reached its lowest level in goats [9]. In microbial ecology, higher alpha diversity typically indicates a broader spectrum of available substrates and a less restrictive environmental filter [2]. The elevated richness and diversity in Simmental beef cattle likely stem from the multi-substrate feeding strategy employed during the growing-finishing stage, where the coexistence of starch-rich grains and mixed forage inputs accommodates multiple functional guilds simultaneously—including cellulolytic pioneers, amylolytic specialists, and cross-feeding lactate utilizers—thereby expanding the spatial and functional niches within the ruminal fluid [24]. Conversely, the significant contraction of microbial diversity observed in lactating Holstein cows highlights the narrowing selective pressure imposed by high-yield dairy management [25]. The metabolic demand for milk production requires highly fermentable, carbohydrate-dense rations that systematically drop the ruminal pH toward the threshold of subacute ruminal acidosis; this chronic micro-acidic state acts as a severe chemical filter, suppressing acid-sensitive fibrolytic taxa while selectively enriching fast-growing, acid-tolerant amylolytic specialists, which ultimately drives a reduction in overall species richness [25]. This filter operates even more strictly in the streamlined ecosystem of the Boer goat. The small rumen volume relative to metabolic body weight, coupled with elevated chewing frequencies per unit of feed, shapes a highly synchronized, non-acidic habitat that restricts the colonization of opportunistic, acidophilic amylolytic species while favoring highly specialized, slow-growing cellulolytic communities, resulting in a condensed but exceptionally efficient core microbiota [8,19].

To resolve whether these structural shifts translate into functional adaptations, the taxonomic enrichment identified through linear discriminant analysis effect size must be contextualized within the host’s metabolic demands and the predictive functional profiling of the metagenome [26]. In Holstein cows, the significant enrichment of the phylum *Proteobacteria* and the genera *Succiniclasticum*, *Succinivibrionaceae_UCG-001*, and *Prevotella_7* represents a clear alignment with the metabolic precursors required for lactation [16,22]. High-producing dairy cows cannot absorb adequate glucose directly from the small intestine; instead, the glucose required by the mammary gland for lactose synthesis must be de novo synthesized in the liver via gluconeogenesis, with ruminal propionate serving as the predominant substrate [17]. Members of the *Succinivibrionaceae* family and *Succiniclasticum* specialize in the succinate pathway, effectively converting soluble carbohydrates or intermediate metabolites into succinate and subsequently decarboxylating it to produce propionate [27]. This metabolic orientation is strongly corroborated by the significant enrichment of ATP-binding cassette transporters in Holstein cows, which facilitates the active transport and rapid uptake of monosaccharides and amino acids across microbial membranes to match the high nutrient density and rapid turnover of lactation diets. Conversely, Simmental beef cattle preferentially enrich *Succinivibrionaceae_UCG-002*, *Ruminococcus_2*, and the *Ruminococcaceae_NK4A214_group*, alongside an upregulation of the glycolysis and gluconeogenesis pathway [28]. This dual enrichment ensures that while fibrolytic specialists aggressively break down the structural carbohydrate fractions of the forage, the core microbiota accelerates the processing of non-structural carbohydrates into pyruvate and acetyl-coenzyme A, optimizing volatile fatty acid generation to support skeletal muscle hypertrophy and intramuscular fat deposition [26]. In sharp contrast, the Boer goat microbiota bypasses this carbohydrate-heavy model, instead enriching the *norank_f_Bacteroidales_BS11_gut_group* and *Lachnospiraceae_ND3007_group* alongside a marked upregulation of oxidative phosphorylation and prokaryotic carbon fixation pathways [29]. This metabolic signature indicates that under high-fiber, lower-energy dietary regimens, the caprine microbiota relies heavily on high-efficiency, baseline adenosine triphosphate generating pathways and autotrophic or mixotrophic carbon fixation to maximize microbial biomass synthesis and maintain ecosystem homeostasis, demonstrating an evolutionary focus on nutritional resilience rather than rapid substrate throughput [30].

These functional differences are directly responsible for shaping the physiological fermentation phenotypes observed across the three host species, establishing a clear causal link within the rumen-fermentation-microbiota axis [31]. The high molar proportion of acetate and elevated acetate-to-propionate ratio in Boer goats reflect a classic acetate-type fermentation model driven by the enrichment of the *norank_f_Bacteroidales_BS11_gut_group* and *Lachnospiraceae_ND3007_group* [32]. The strong positive correlation of these specific taxa with both pH and the acetate-to-propionate ratio underscores their role in maintaining fiber-driven fermentation profiles. In contrast, the high-grain input in Simmental cattle enriches *Succinivibrionaceae_UCG-002*, which correlates positively with propionate production and negatively with ruminal pH, driving a propionate-dominated fermentation profile. By shifting the fermentation kinetics toward propionate, the micro-ecosystem optimizes energy retention for the host, channeling key carbon skeletons toward hepatic glucose synthesis and subsequent muscle tissue growth [33]. Concurrently, the unique nitrogen dynamics observed in Holstein cows, which exhibited the lowest ruminal ammonia nitrogen concentration, further illustrate host-microbe coordination [34]. This low concentration reflects an exceptional efficiency in microbial nitrogen incorporation rather than a deficiency in degradable protein. The high availability of readily fermentable energy from starch synchronizes with nitrogen release, allowing dominant taxa like *Succinivibrionaceae_UCG-001* and *Prevotella_7* to rapidly utilize free ammonia for microbial crude protein synthesis, thereby maximizing the supply of high-quality amino acids entering the duodenum for milk protein synthesis [35].

From an eco-physiological and environmental standpoint, the significant downregulation of the methane metabolism pathway in Holstein cows compared to both Simmental cattle and Boer goats provides important insights into the ruminal hydrogen economy [8,36]. Methanogenesis represents a vital thermodynamic necessity in the rumen, serving to dispose of metabolic hydrogen or molecular hydrogen generated during carbohydrate fermentation and preventing the feedback inhibition of microbial dehydrogenases [36]. However, the combination of a grain-rich dairy diet and a *Succinivibrionaceae_UCG-001*-dominated flora type fundamentally alters this pathway [37]. Because *Succinivibrionaceae* utilize the competitive succinate pathway to generate propionate, they have the computational potential to function as alternative hydrogen sinks, possibly competing with methanogenic archaea for the available metabolic hydrogen pool [38]. This predicted competition might divert hydrogen flux away from the canonical carbon-dioxide reduction pathway that drives methanogenesis, thereby lowering the overall methane-production potential in the rumen of dairy cows [36]. Nevertheless, since actual in vivo methane emissions were not directly quantified in our present work, such mechanistic inferences remain speculative and need further validation via comprehensive respiratory-chamber trials. This competitive pathway explains the lower metagenomic score for methane metabolism in Holstein cows and offers a valuable natural blueprint for designing targeted micro-ecological interventions aimed at mitigating greenhouse gas emissions without compromising animal productivity [39]. Furthermore, the lower baseline energy metabolism score in Holstein cows fits with the principles of host-microbe energy partitioning under high digesta passage rates, where the microbiota prioritizes the rapid output of volatile fatty acids over its own long-term biomass maintenance, illustrating a highly streamlined, economically optimized symbiotic system [40]. Our prediction of suppressed methane metabolic pathways in Holstein cows offers novel perspectives on potential ruminal hydrogen rebalancing. We hypothesize that members of the *Succinivibrionaceae* may serve as alternative hydrogen sinks to replace methanogens, and such competitive niche partitioning diverts hydrogen from the canonical CO_2_ reduction pathway. Nevertheless, interpretations derived from PICRUSt-based KEGG pathway prediction cannot serve as direct in vivo evidence for actual methane production, and these explanations remain hypothetical descriptions of host-microbe crosstalk. The recent literature has further addressed this limitation: as demonstrated by prior research [41] evaluating methane profiles requires distinguishing among methane yield, total methane production, and residual methane emissions, especially since enhanced feed efficiency does not inherently guarantee reduced daily methane emissions. Furthermore, translating these predicted microbial differences into practical mitigation strategies demands a comprehensive approach. Previous investigations [42] have cautioned that the implementation of methane reduction interventions, such as chemical inhibitors, must be carefully weighed against potential unintended consequences on baseline rumen function and overall animal performance. Ultimately, linking microbial methane metabolism with environmental sustainability necessitates a holistic view of global food systems. Following the perspectives of prior agroecological [43] any methane mitigation effort must be balanced with global food security and the unique evolutionary capacity of ruminants to convert human-inedible biomass into high-quality animal protein, ensuring that environmental targets do not compromise livestock efficiency and nutritional sustainability. Relevant fermentation trials [44] highlighted how shifts in acetate-to-propionate ratios alter ruminal fermentation and methane emissions, while systematic reviews [45] comprehensively summarized the contributions of rumen microbiota and metabolic pathways to enteric methane mitigation. These studies emphasize the necessity of cautious interpretation of metagenomic methane-related profiles. In addition, the significant Spearman’s correlations reported herein only demonstrate statistical covariation and cannot confirm direct biological regulation or functional interactions between variables.

Ultimately, the multidimensional correlation network generated in this study integrates these individual components into a cohesive regulatory axis linking core microbiota, metabolic pathways, and fermentation phenotypes, demonstrating that the ruminal response is driven by coordinated shifts across the entire community rather than isolated bacterial genera [15,46]. Prevotella_1 emerges as a foundational generalist across both bovine species, maintaining widespread positive correlations with nitrogen metabolism, carbon fixation, energy metabolism, and the production of both acetate and propionate [2]. This broad functional profile indicates that Prevotella_1 provides vital functional redundancy, ensuring the stability of the bovine rumen when dietary compositions change [46]. Conversely, the specialized, distinct correlation profiles of *Succinivibrionaceae_UCG-001* and *Succinivibrionaceae_UCG-002* clarify the practical consequences of host-driven flora-typing, showing how *UCG-001* adapts to high-throughput carbohydrate fermentation during lactation while *UCG-002* links with a wider range of biosynthetic pathways to support beef cattle growth. On the small ruminant axis, the strong positive correlation of the *norank_f_Bacteroidales_BS11_gut_group* and *Lachnospiraceae_ND3007_group* with butyrate production highlights a host-specific adaptation for gastrointestinal health [47]. Because butyrate serves as the primary energy source for the ruminal epithelium and stimulates papillary development, the selective enrichment of butyrate-producing taxa in goats highlights a mechanism by which small ruminants maintain mucosal barrier integrity and enhance nutrient absorption under high-fiber feeding conditions [48].

The clear separation of ruminal microbial structures observed across the three livestock populations provides robust observational evidence that host developmental trajectories, selection history and species-specific feeding regimes synergistically shape rumen microecological divergence. Nevertheless, intrinsic disparities among species in age, sex, specific physiological stages (such as mid-lactation females versus growing males), production objectives, dietary composition and farm environments preclude exclusive attribution of observed differences to host genetics. The detected community divergence instead reflects the combined, interrelated effects of the whole production system rather than isolated host genetic factors.

## 5. Conclusions

**Figure 5:**
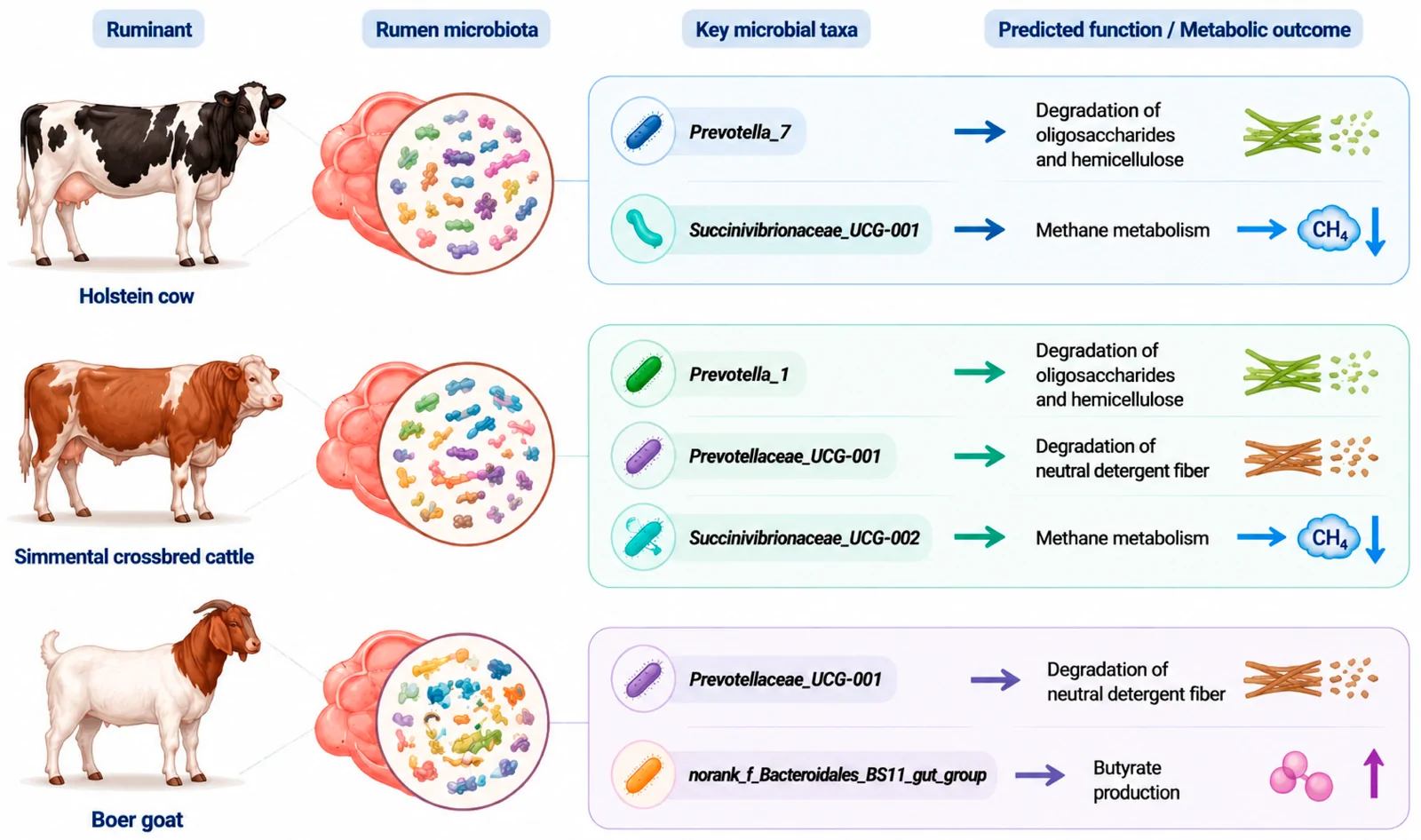
Relationship between rumen microbiota, microbial metabolism pathways and rumen fermentation parameters, where upward-pointing arrows indicate an increase and downward-pointing arrows indicate a decrease.

This study demonstrates specific interactive relationships among host ruminant species, rumen microbiota, metabolic pathways, and fermentation parameters (Figure 5). Notably, microbes associated with oligosaccharide and hemicellulose degradation (e.g., Prevotella) are more abundant in Holstein cows and Simmental crossbred cattle than in Boer goats. While Holstein cows exhibit a lower proportion of neutral detergent fiber-degrading taxa (*Prevotellaceae_UCG-001*), they harbor the highest relative abundance of *Proteobacteria*, a phylum closely associated with milk yield. Furthermore, the synthesis of short-chain fatty acids (SCFAs) in Holstein cows, Simmental cattle, and Boer goats is distinctly driven by the metabolic pathways of *Succinivibrionaceae_UCG-001*, *Succinivibrionaceae_UCG-002*, and *Lachnospiraceae_ND3007_group*, respectively. In Boer goats, this SCFA production is uniquely accompanied by the exclusive enrichment of the *norank_f_Bacteroidales_BS11_gut_group* and a significant positive correlation between the *Lachnospiraceae_ND3007_group* and methane metabolic pathways.

Collectively, these findings provide critical theoretical foundations for targeted modulation of the ruminal microbiome to improve feed utilization efficiency, optimize host health status, and reduce enteric methane emissions in diverse ruminant breeding systems. Overall, this study established a correlational analytical framework that characterizes the fundamental interaction patterns of ruminal microbial communities across various ruminant production systems and identifies predictive correlations between specific enterotypes and methane metabolic pathways. Nevertheless, this research only demonstrates statistical correlations between microbial enterotypes and methane pathways; in vivo functional trials are still required to further verify the practical application efficacy of the proposed regulatory strategy.

## Figures and Tables

**Table 1:** Rumen fermentation parameters of Holstein cows, Simmental crossbred cattle and Boer goats.

Items	Treatments		
	Holstein Cows	Simmental Crossbred Cattle	Boer Goats
pH	6.42 ± 0.07 b	6.28 ± 0.03 c	6.5 ± 0.14 a
NH3-N (mg/dL)	12.11 ± 1.17 c	16.99 ± 0.52 a	13.41 ± 0.86 b
Acetate (%)	63.75 ± 1.14 a	60.77 ± 0.79 c	61.68 ± 1.65 b
Propionate (%)	24.82 ± 1.03 b	26.97 ± 0.55 a	21.19 ± 0.91 c
Butyrate (%)	11.43 ± 0.69 b	12.26 ± 0.48 a	12.01 ± 0.58 a
Acetate/Propionate	2.57 ± 0.14 b	2.24 ± 0.1 c	2.92 ± 0.16 a

Values are presented as means ± standard error. Superscript letters (a, b, c) within the same row indicate significant pairwise differences. Statistical significance was determined using the Kruskal–Wallis H test followed by Wilcoxon rank-sum post hoc tests with False Discovery Rate (FDR) adjustment (*p* < 0.05).

## Data Availability

According to the requirements, data can be obtained from the corresponding authors.

## Supplementary Materials

The following supporting information can be downloaded at: https://www.mdpi.com/article/10.3390/microorganisms14091935/s1, Figure S1. Rumen microbial composition of Holstein cows (HC), Simmental crossbred cattle (SCC), and Boer goats (BG) at phylum (A) and genus (B) levels. Horizontal coordinate is the sample name, vertical coordinate is the proportion of microbiota in that sample, the different coloured columns represent different microbiota, and the length of the column represents the size of the proportion of that species. Figure S2. Rumen microbiota functional prediction of genus-level Holstein cows (HC) and Simmental crossbred cattle (SCC) (A), Holstein cows (HC) and Boer goats (BG) (B), Simmental crossbred cattle (SCC) and Boer goats (BG) (C) rumen microbiota using KEGG pathway level 1 level. The *y*-axis indicates the name of a metabolic pathway, the *x*-axis indicates the average relative abundance in different groupings of species, and the different coloured columns indicate the different ruminant groupings. Values on the right are *p*-values indicating significance, * *p* < 0.05, ** *p* < 0.01, *** *p* < 0.001. Figure S3. Rumen microbiota functional prediction of genus-level Holstein cows (HC) and Simmental crossbred cattle (SCC) rumen microbiota using KEGG pathway level 3 level. The *y*-axis indicates the name of a metabolic pathway, the *x*-axis indicates the average relative abundance in different groupings of species, and the different coloured columns indicate the different ruminant groupings. Values on the right are *p*-values indicating significance, * *p* < 0.05, ** *p* < 0.01, *** *p* < 0.001. Figure S4. Rumen microbiota functional prediction of genus-level Holstein cows (HC) and Boer goats (BG) rumen microbiota using KEGG pathway level 3 level. The *y*-axis indicates the name of a metabolic pathway, the *x*-axis indicates the average relative abundance in different groupings of species, and the different coloured columns indicate the different ruminant groupings. Values on the right are *p*-values indicating significance, * *p* < 0.05, ** *p* < 0.01, *** *p* < 0.001. Figure S5. Rumen microbiota functional prediction of genus-level Simmental crossbred cattle (SCC) and Boer goats (BG) rumen microbiota using KEGG pathway level 3 level. The *y*-axis indicates the name of a metabolic pathway, the *x*-axis indicates the average relative abundance in different groupings of species, and the different coloured columns indicate the different ruminant groupings. Values on the right are *p*-values indicating significance, * *p* < 0.05, ** *p* < 0.01, *** *p* < 0.001. Figure S6. Heat map of correlation between rumen microbiota (genus level) and KEGG pathway level 3 microbial metabolic pathway in Holstein cows. Horizontal coordinates are microbial metabolic pathway names, vertical coordinates are microbial names, and shades of color indicate data values. Asterisks indicate statistical significance; * 0.01 < *p* ≤ 0.05, ** 0.001 < *p* ≤ 0.01, *** *p* ≤ 0.001. Figure S7. Heat map of correlation between rumen microbiota (genus level) and KEGG pathway level 3 microbial metabolic pathway in Simmental crossbred cattle. Horizontal coordinates are microbial metabolic pathway names, vertical coordinates are microbial names, and shades of color indicate data values. Asterisks indicate statistical significance; * 0.01 < *p* ≤ 0.05, ** 0.001 < *p* ≤ 0.01, *** *p* ≤ 0.001. Figure S8. Heat map of correlation between rumen microbiota (genus level) and KEGG pathway level 3 microbial metabolic pathway in Boer goats. Horizontal coordinates are microbial metabolic pathway names, vertical coordinates are microbial names, and shades of color indicate data values. Asterisks indicate statistical significance, * *p* < 0.05, ** *p* < 0.01, *** *p* < 0.001. Figure S9. Analysis of the correlation between rumen microbiota, microbial metabolism pathway and rumen fermentation parameters. Red represents positive correlation, and blue represents negative correlation. Table S1. Diet composition and nutritional level of Holstein cows. Table S2. Diet composition and nutritional level of Simmental crossbred cattle. Table S3. Diet composition and nutritional level of Boer goats.
