# Supplementary material for: Rumen Microbiota, Predicted Metabolic Pathways, and Fermentation Parameters Exhibit Production-System-Specific Associations in Cattle and Goats
Source: Microorganisms. 2026 Sep 1;14(9):1935. doi: 10.3390/microorganisms14091935 (PMC13609192; doi:10.3390/microorganisms14091935)
Supplement: Supplementary file 1 [file microorganisms-14-01935-s001.zip › microorganisms-4502112-supplementary.pdf]

Supplementary Materials

Supplementary Figures

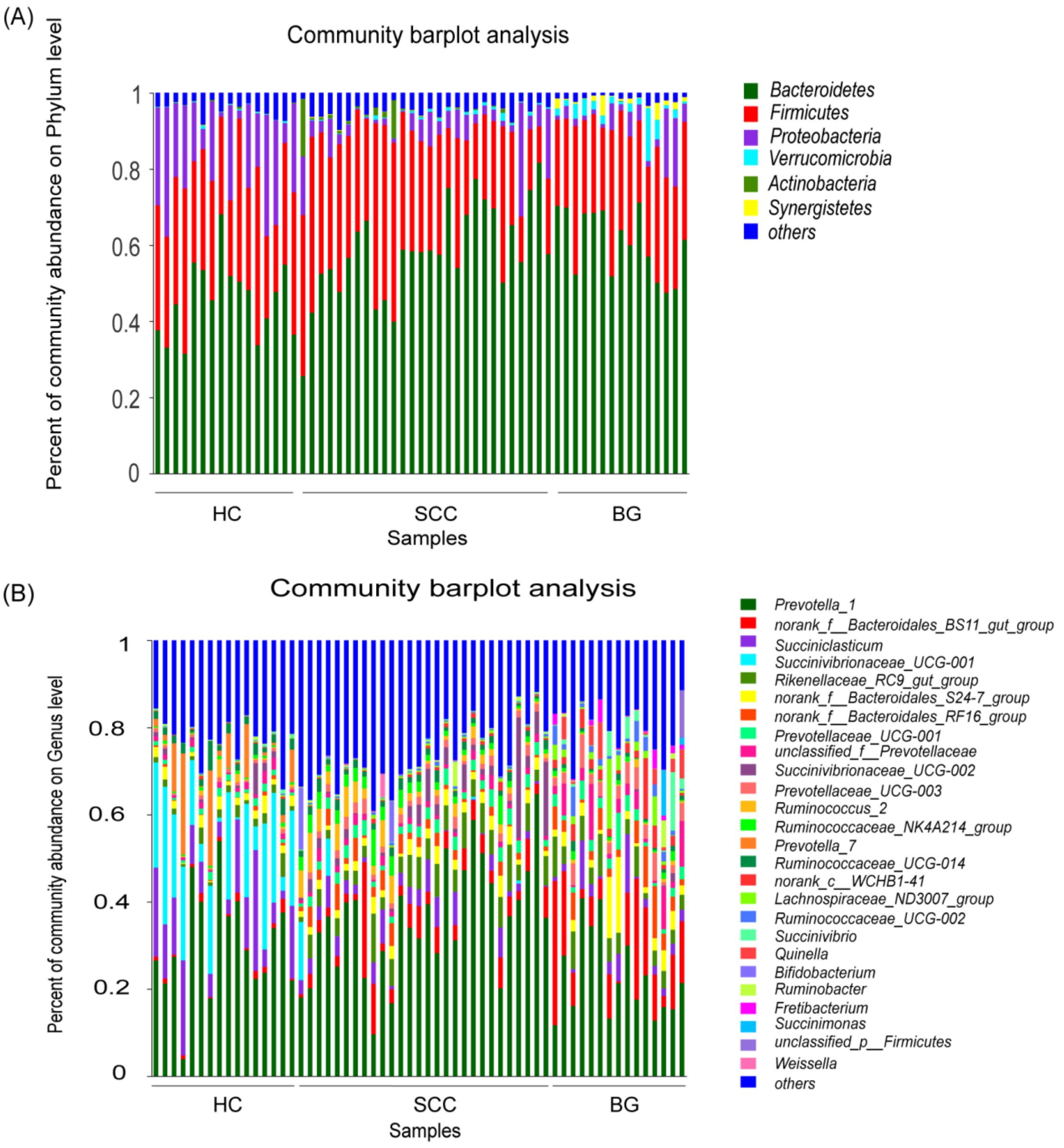

Fig. S1 Rumen microbial composition of Holstein cows (HC), Simmental crossbred cattle (SCC), and Boer goats (BG) at phylum (A) and genus (B) levels. Horizontal coordinate is the sample name, vertical coordinate is the proportion of microbiota in that sample, the different coloured columns represent different microbiota, and the length of the column represents the size of the proportion of that species.

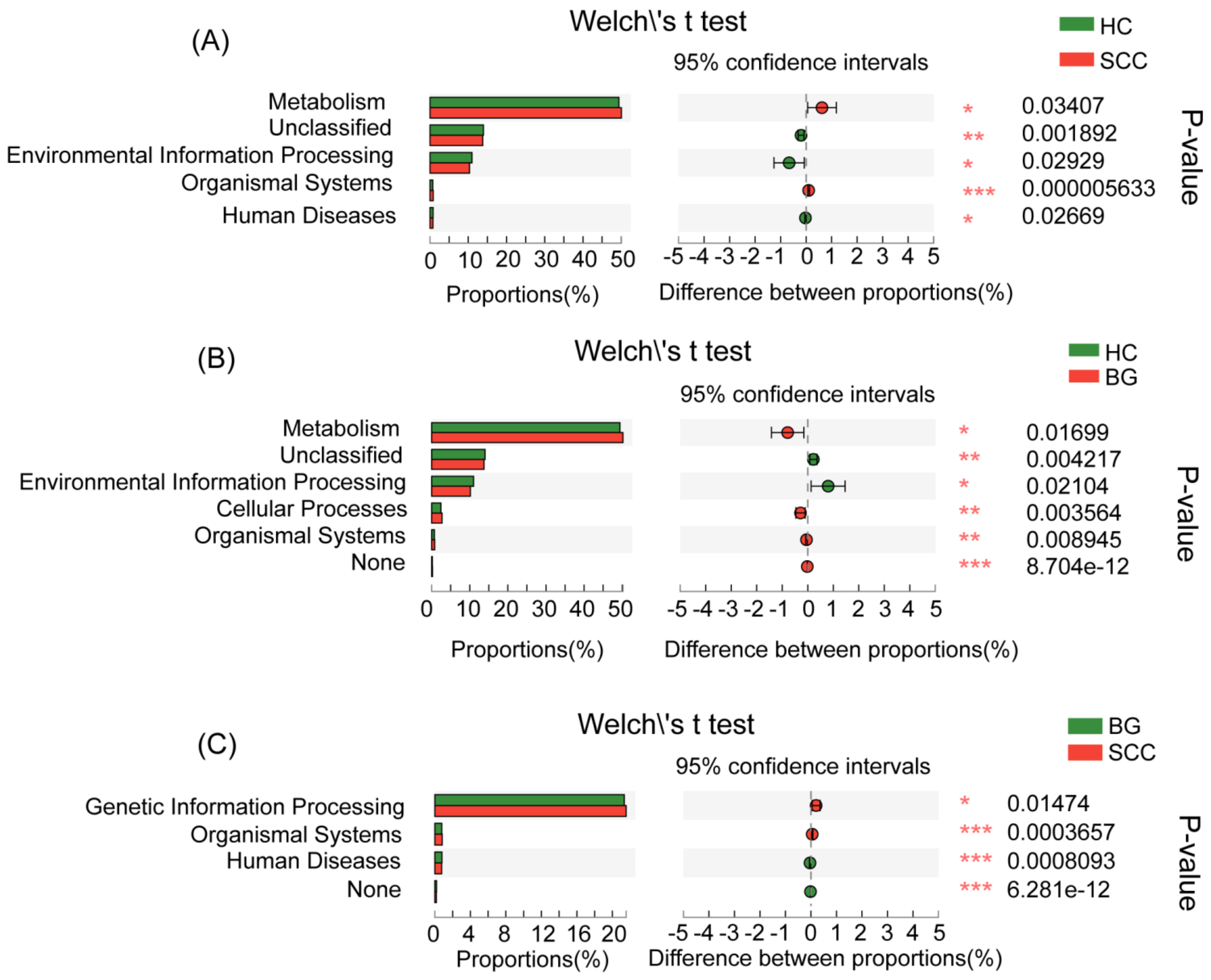

Fig. S2 Rumen microbiota functional prediction of genus-level Holstein cows (HC) and Simmental crossbred cattle (SCC) (A), Holstein cows (HC) and Boer goats (BG) (B), Simmental crossbred cattle (SCC) and Boer goats (BG) (C) rumen microbiota using KEGG pathway level 1 level. The y-axis indicates the name of a metabolic pathway, the x-axis indicates the average relative abundance in different groupings of species, and the different coloured columns indicate the different ruminant groupings. Values on the right are *P*-values indicating significance, * $P < 0.05$, ** $P < 0.01$, *** $P < 0.001$.

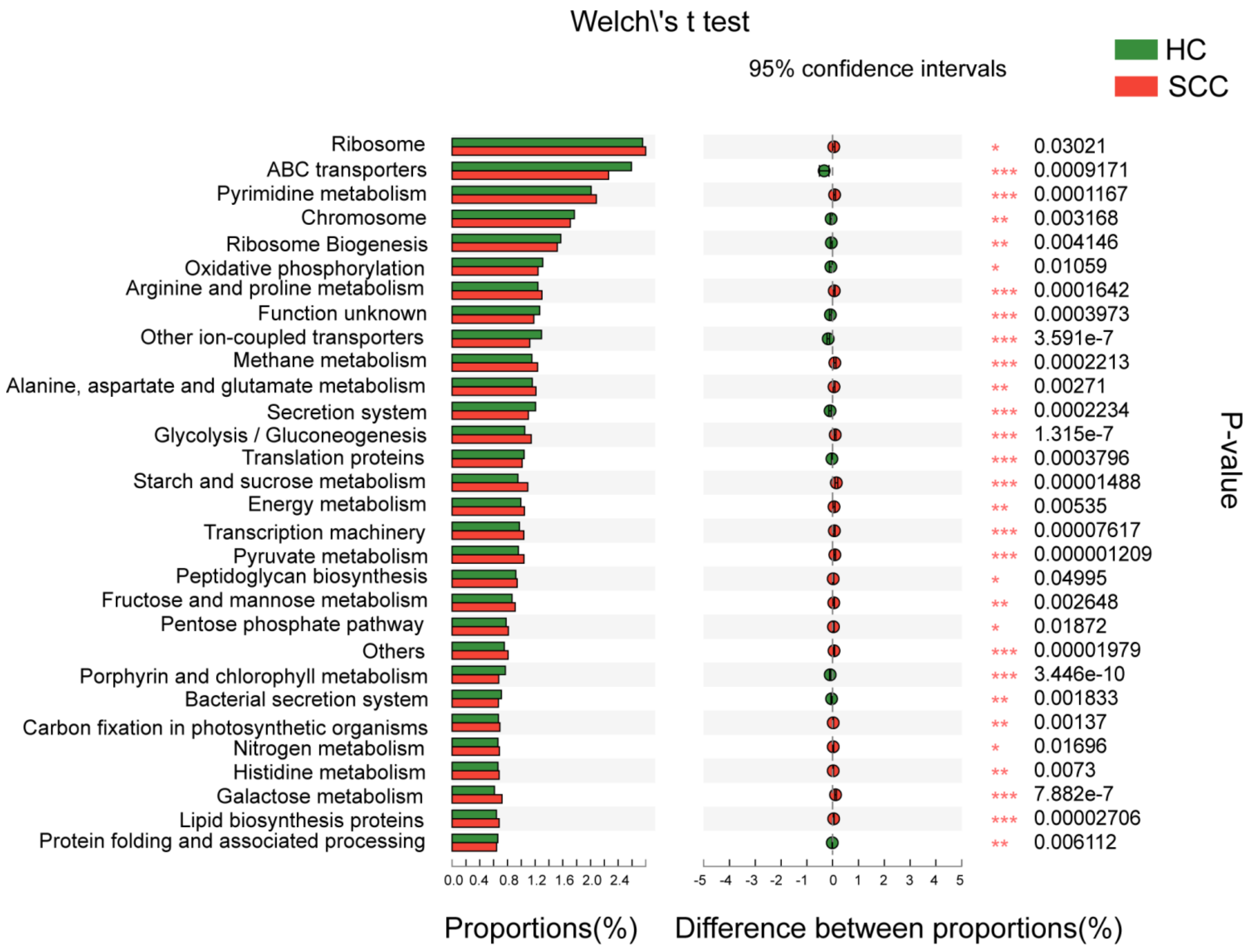

Fig. S3 Rumen microbiota functional prediction of genus-level Holstein cows (HC) and Simmental crossbred cattle (SCC) rumen microbiota using KEGG pathway level 3 level. The y-axis indicates the name of a metabolic pathway, the x-axis indicates the average relative abundance in different groupings of species, and the different coloured columns indicate the different ruminant groupings. Values on the right are *P*-values indicating significance, * $P < 0.05$, ** $P < 0.01$, *** $P < 0.001$.

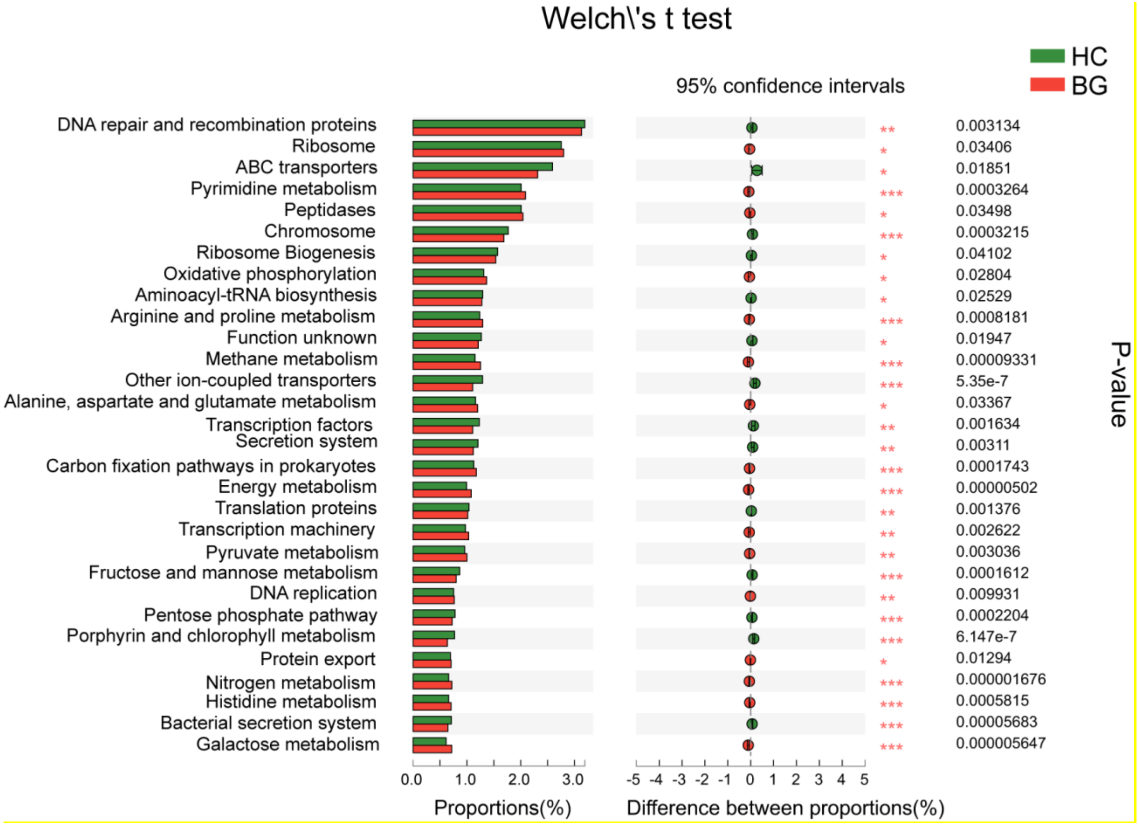

Fig. S4 Rumen microbiota functional prediction of genus-level Holstein cows (HC) and Boer goats (BG) rumen microbiota using KEGG pathway level 3 level. The y-axis indicates the name of a metabolic pathway, the x-axis indicates the average relative abundance in different groupings of species, and the different coloured columns indicate the different ruminant groupings. Values on the right are *P*-values indicating significance, * $P < 0.05$, ** $P < 0.01$, *** $P < 0.001$.

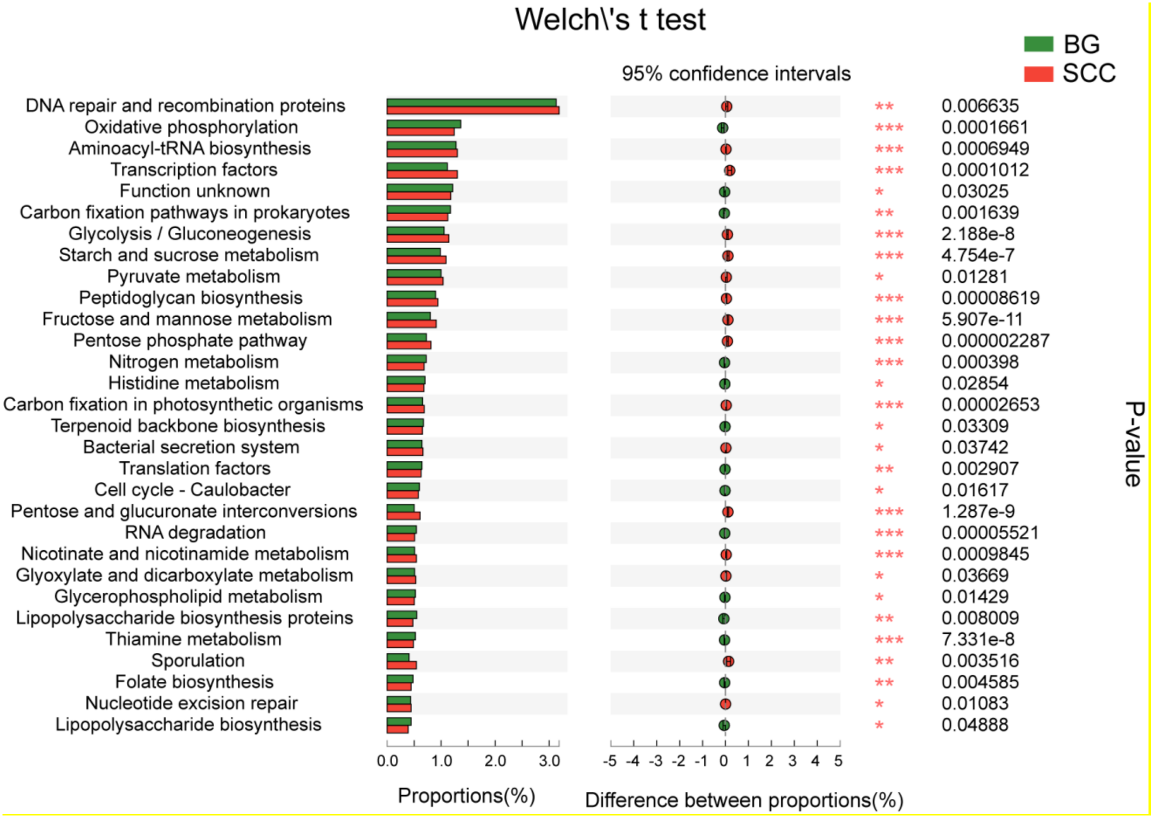

Fig. S5 Rumen microbiota functional prediction of genus-level Simmental crossbred cattle (SCC) and Boer goats (BG) rumen microbiota using KEGG pathway level 3 level. The y-axis indicates the name of a metabolic pathway, the x-axis indicates the average relative abundance in different groupings of species, and the different coloured columns indicate the different ruminant groupings. Values on the right are *P*-values indicating significance, * $P < 0.05$, ** $P < 0.01$, *** $P < 0.001$.

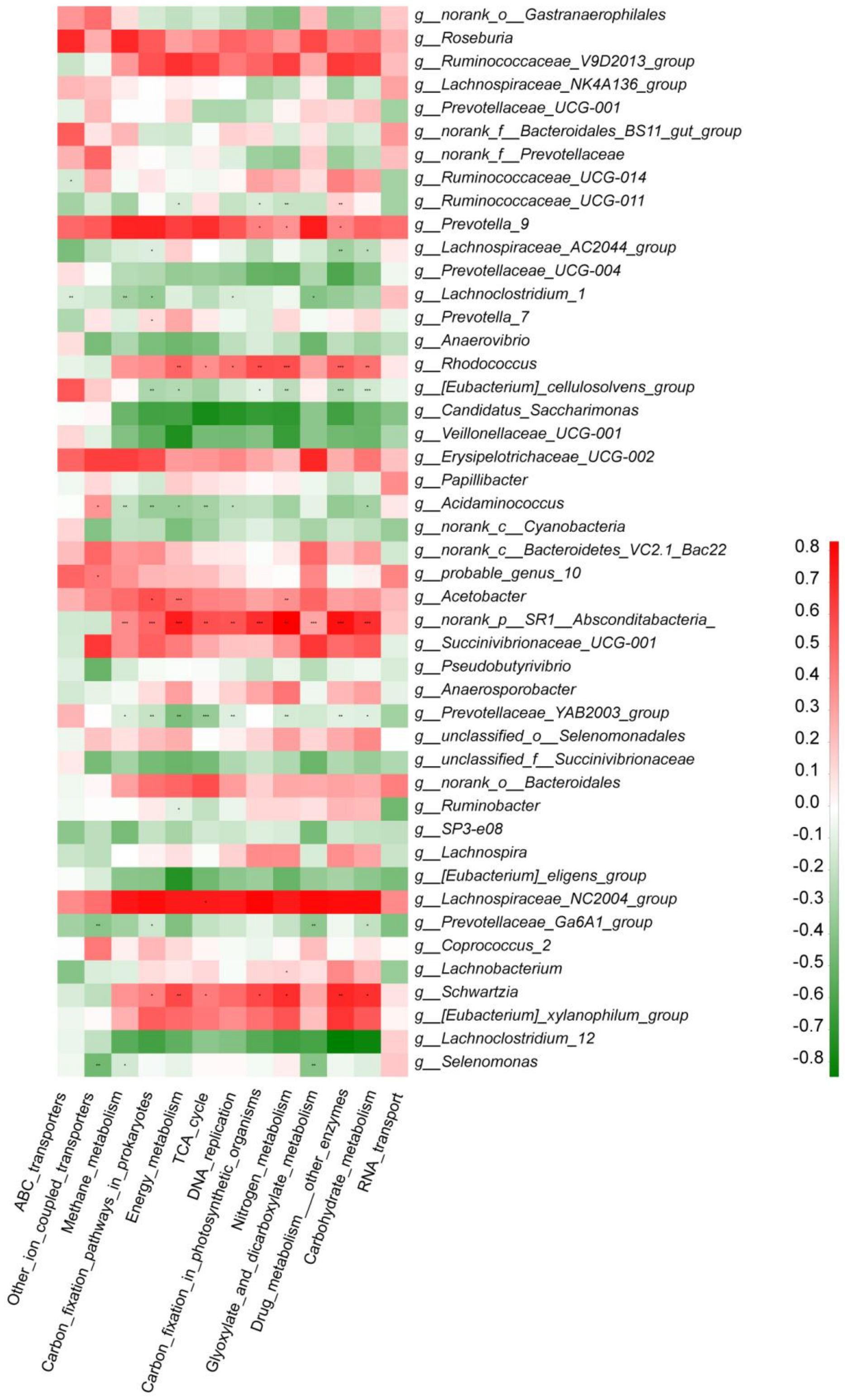

Fig. S6 Heat map of correlation between rumen microbiota (genus level) and KEGG pathway level 3 microbial metabolic pathway in Holstein cows. Horizontal coordinates are microbial metabolic

pathway names, vertical coordinates are microbial names, and shades of color indicate data values. Asterisks indicate statistical significance; * 0.01 < $P \leq 0.05$, ** 0.001 < $P \leq 0.01$, *** $P \leq 0.001$.

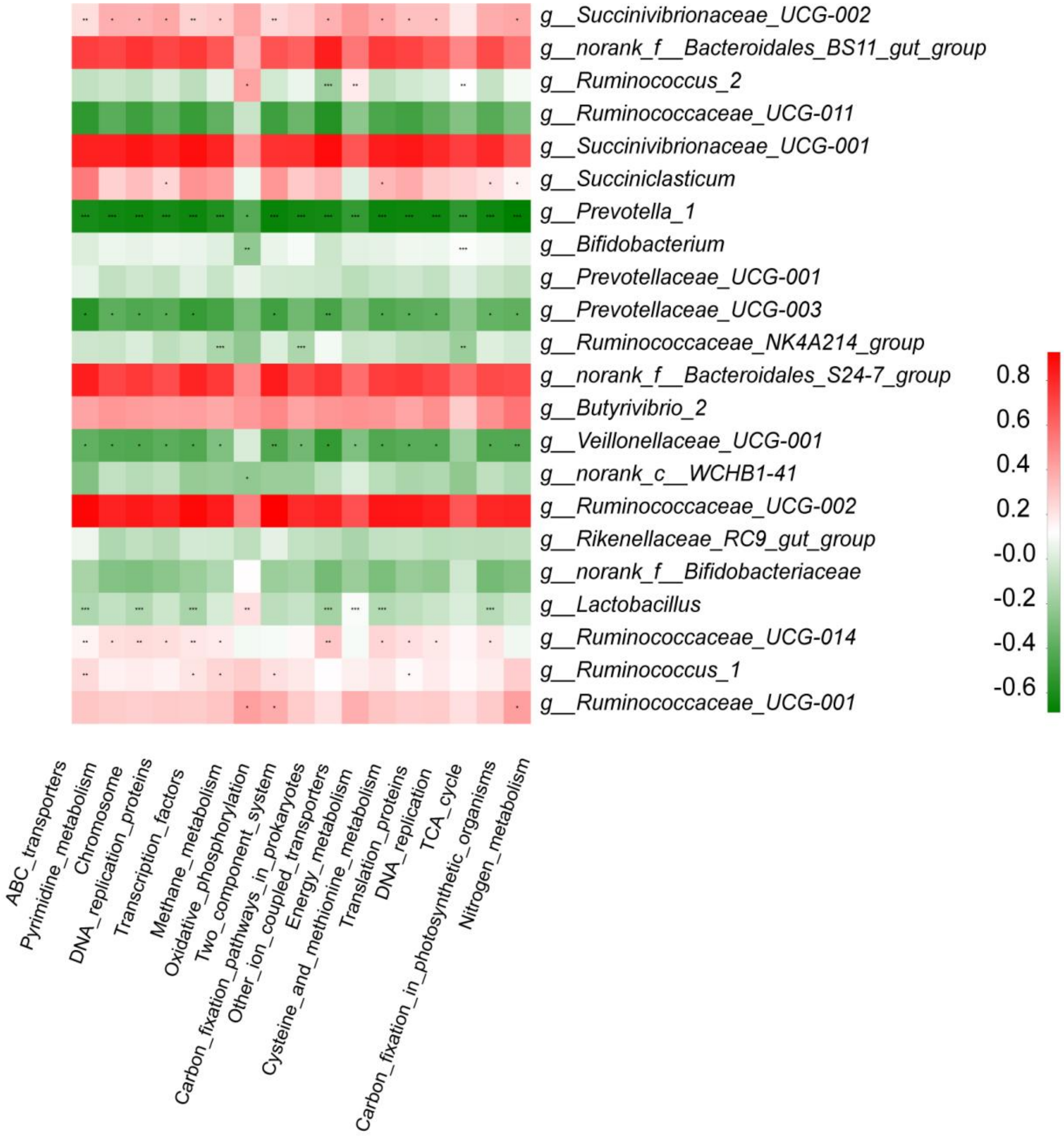

Fig. S7 Heat map of correlation between rumen microbiota (genus level) and KEGG pathway level 3 microbial metabolic pathway in Simmental crossbred cattle. Horizontal coordinates are microbial metabolic pathway names, vertical coordinates are microbial names, and shades of color indicate data values. Asterisks indicate statistical significance; * 0.01 < $P \leq 0.05$, ** 0.001 < $P \leq 0.01$, *** $P \leq 0.001$.

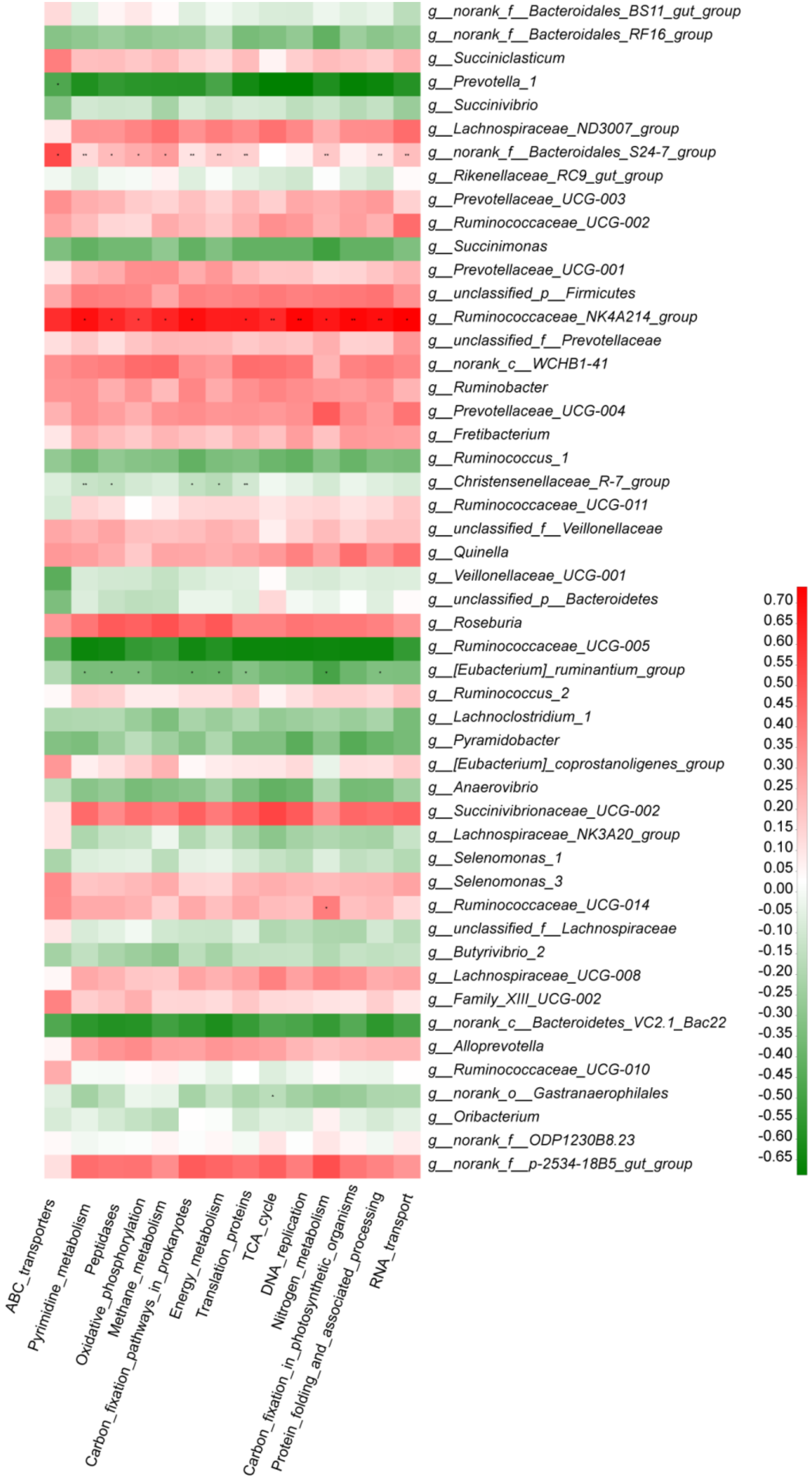

Fig. S8 Heat map of correlation between rumen microbiota (genus level) and KEGG pathway level 3 microbial metabolic pathway in Boer goats. Horizontal coordinates are microbial metabolic pathway names, vertical coordinates are microbial names, and shades of color indicate data values. Asterisks

indicate statistical significance, * $P < 0.05$, ** $P < 0.01$, *** $P < 0.001$.

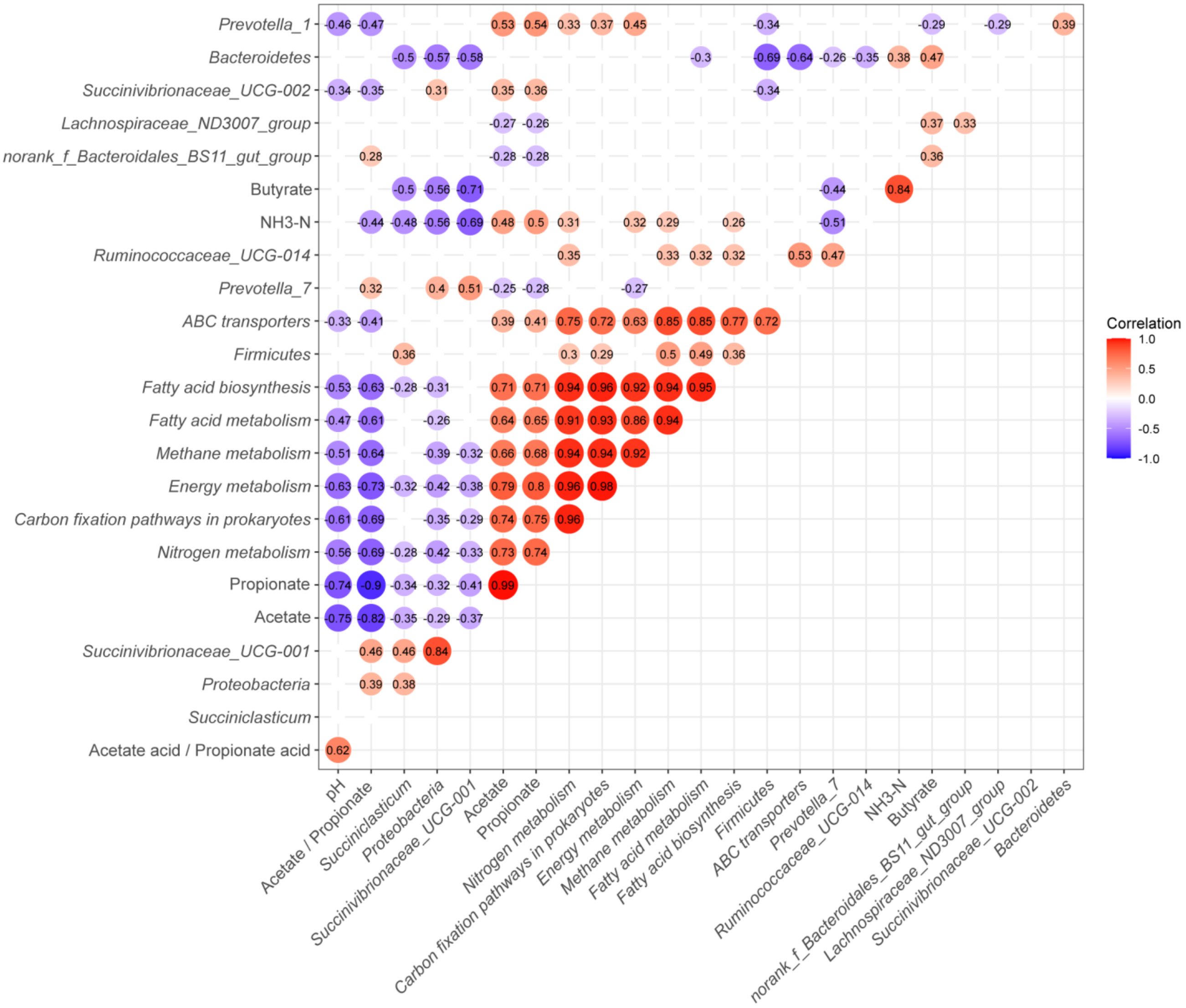

Fig. S9 Analysis of the correlation between rumen microbiota, microbial metabolism pathway and rumen fermentation parameters. Red represents positive correlation, and blue represents negative correlation.

2. Supplementary Tables

Table S1. Diet composition and nutritional level of Holstein cows

**Diet composition**	
Alfalfa (%)	13.23
Corn silage (%)	24.01
Chinese hay (%)	13.23
Mixed concentrate (%) [1]	49.53
Total (%)	100
Concentrate/roughage	1.00
**Nutrient level [2]**	
NE_L (MJ/kg)	6.99
CP (%)	15.20
NDF (%)	35.27
ADF (%)	20.08
Ca (%)	0.55
P (%)	0.32

[1] Mixed concentrate: corn = 29.0%, corn compressor = 28.0%, soybean meal = 12.0%, corn DDGS = 12.0% cottonseed meal = 12.0%, $CaHPO_4$ = 2.0% $CaCO_3$ = 0.7%, NaCl = 1.0% $NaHCO_3$ = 1.5%, ruminant-specified active yeast feed (XP) = 0.5%, MgO = 0.5%, premix = 0.8%. One kilogram of premix contains the following: $CuSO_4 \cdot 5H_2O$ = 3,125 mg, $FeSO_4 \cdot H_2O$ = 9,375mg, $MnSO_4 \cdot H_2O$ = 14,375 mg, $ZnSO_4 \cdot 7H_2O$ = 13,125 mg, Co = 0.3 mg, Se = 0.2 mg, I (as potassium iodide) = 6.25 mg, Vitamin A = 1,500,000 IU, Vitamin D_3 = 1,250,000 IU, Vitamin E = 3,125mg, niacin =4,500 mg , and choline = 125,000 mg.

[2] Nutrient level: CP = crude protein, NDF = neutral detergent fiber, Ca = calcium, and P = phosphorus. NF_L are calculated values, while other nutrient levels are measure values.

Table S2. Diet composition and nutritional level of Simmental crossbred cattle

**Diet composition**	
Silage corn (%)	40.00
Wheat straw (%)	10.00
Mixed concentrate (%) [1]	50.00
Total (%)	100
Concentrate/roughage	1.00
**Nutrient level[3]**	
Nemf (MJ/kg)	9.25
CP (%)	16.54
NDF (%)	35.94
ADF (%)	20.27
Ca (%)	0.40
P (%)	0.41

[1] Mixed concentrate: corn = 50.0%, bran = 15.0%, soybean meal = 16.0%, cottonseed meal = 6.0%, Rapeseed meal = 4.8%, Limestone = 2.0%, $CaHPO_4$ = 0.2%, NaCl = 1.0%, Premix (%) [2] = 5.0%.

[2] Premix (per kilogram of feed content): Vitamin A = 2,640 IU, Vitamin D_3 = 340 IU, Vitamin E = 26 mg, Fe = 60 mg, Cu = 12 mg, Zn = 48 mg,Go = 0.12 mg, I = 0.3 mg, and Se = 0.36 mg.

[3] Nutrient level: CP = crude protein, NDF = neutral detergent fiber, ADF = acid detergent fiber, Ca = calcium, and P = phosphorus. The Nemf in the table was calculated and the rest of the nutrition index were measured.

Table S3. Diet composition and nutritional level of Boer goats

**Diet composition**	
Cassava stem and leaf (%)	50.00
Mixed concentrate (%) [1]	50.00
Total (%)	100
Concentrate/roughage	1.00
**Nutrient level [3]**	
CP (%)	16.67
NDF (%)	33.93
ADF (%)	20.92
Ca (%)	0.95
P (%)	0.43

[1] Mixed concentrate: corn = 33.50%, wheat meal = 4.90%, soybean meal = 9.00%, conch meal = 0.70% sodium bicarbonate = 0.30% NaCl = 0.70% $CaHCO_3$ = 0.40%, premix (%) [2] = 0.50%.

[2] Premix provided the following per kilogram of diets: Vitamin A = 15,000 IU, Vitamin D_3 = 5,000 IU, Vitamin E = 50 mg, Iron = 9 mg, Zinc = 100 mg, Mn = 130 mg, Selenium = 0.3 mg, Iodine = 36 mg.

[3] Nutrient level: DE = digestible energy, CP = crude protein, NDF = neutral detergent fiber, ADF = acid detergent fiber, Ca = calcium, and P = phosphorus. The levels of CP, NDF and ADF were measured values, while Ca and P were calculated values.
